# Regulation of the phototrophic iron oxidation (*pio*) genes in *Rhodopseudomonas palustris* TIE-1 is mediated by the global regulator, FixK

**DOI:** 10.1111/j.1365-2958.2010.07430.x

**Published:** 2010-10-28

**Authors:** Arpita Bose, K Newman Dianne

**Affiliations:** Departments of 1 Biology and; 3 Earth, Atmospheric and Planetary Sciences; 2 Massachusetts Institute of Technology, Howard Hughes Medical Institute, 77 Massachusetts Ave., 68-380, Cambridge, MA 02139, USA

## Abstract

The *pioABC* operon is required for phototrophic iron oxidative (photoferrotrophic) growth by the αproteobacterium *Rhodopseudomonas palustris* TIE-1. Expression analysis of this operon showed that it was transcribed and translated during anaerobic growth, upregulation being observed only under photoferrotrophic conditions. Very low levels of transcription were observed during aerobic growth, suggesting expression was induced by anoxia. The presence of two canonical FixK boxes upstream of the identified *pioABC* transcription start site implicated FixK as a likely regulator. To test this possibility, a *δfixK* mutant of *R. palustris* TIE-1 was assessed for *pioABC* expression. *pioABC* expression decreased dramatically in *δfixK* versus WT during photoferrotrophic growth, implying that FixK positively regulates its expression; coincidently, the onset of iron oxidation was prolonged in this mutant. In contrast, *pioABC* expression increased in *δfixK* under all non-photoferrotrophic conditions tested, suggesting the presence of additional levels of regulation. Purified FixK directly bound only the proximal FixK box in gel mobility-shift assays. Mutant expression analysis revealed that FixK regulates anaerobic phototrophic expression of other target genes with FixK binding sites in their promoters. This study shows that FixK regulates key iron metabolism genes in an αproteobacterium, pointing to a departure from the canonical Fur/Irr mode of regulation.

## Introduction

Phototrophic iron oxidation (photoferrotrophy) is a microbial metabolism that was initially described in 1993 and the first photoferrotroph was isolated in 1994 (Widdel *et al*., 1993; Ehrenreich and Widdel, 1994). It involves the light-dependent oxidation of reduced ferrous iron Fe(II) to ferric iron Fe(III) under anoxic conditions, leading to the fixation of carbon dioxide (CO_2_). Currently, photoferrotrophy is known to be performed by five anoxygenic purple (non) sulphur bacteria and one green sulphur bacterium (Ehrenreich and Widdel, 1994; Heising and Schink, 1998; Heising *et al*., 1999; Straub *et al*., 1999; Jiao *et al*., 2005). The interest in photoferrotrophy arose due to its possible role in the deposition of some of the earliest Precambrian banded iron formations (BIFs) (Hartman, 1984; Widdel *et al*., 1993; Croal *et al*., 2004; Weber *et al*., 2006). Although over a decade has passed since the isolation of the first photoferrotroph, very little is known about their cellular and molecular biology. The dearth of tools available to study these organisms has hindered scientists from assessing their importance in modern environments, much less being able to critically speculate on whether ancient BIF deposition was linked to photoferrotrophy. Recent advances in the genetics of *Rhodopseudomonas palustris* TIE-1, a facultative photoferrotroph, have allowed us to at least begin understanding this novel metabolism (Jiao *et al*., 2005; Jiao and Newman, 2007).

*Rhodopseudomonas palustris* TIE-1 is a Gram-negative αproteobacterium (*Rhizobiales*) that was isolated from an iron-rich mat from School Street Marsh in Woods Hole, MA, USA (Jiao *et al*., 2005). This purple non-sulphur bacterium is genetically tractable and a number of tools available for purple phototrophs operate efficiently in this isolate. It is also metabolically versatile, being able to grow aerobically as a chemoheterotroph and anaerobically as a phototroph (Jiao and Newman, 2007). In addition, its genome sequence and those of closely related strains such as *R. palustris* CGA009, *R. palustris* BisB18 and *R. palustris* BisA53 are readily available (Larimer *et al*., 2004; Oda *et al*., 2008, http://genome.ornl.gov/microbial/rpal_tie1/). Using markerless deletion, we showed that the *pioABC* operon of *R. palustris* TIE-1 (Fig. S1) is the genetic locus that allows this organism to perform photoferrotrophy (Jiao and Newman, 2007). PioA is predicted to be a decahaem cytochrome, PioB is a predicted outer membrane porin and PioC is a predicted high potential iron sulphur protein. However, the mechanism of electron transfer from Fe(II) to the reaction centre is not fully understood. Presumably, cyclic electron flow generates ATP, as is known to be the case in other purple phototrophs (Feniouk and Junge, 2009) and reducing equivalents for the formation of NADH derive from reverse electron transport, as occurs in Fe(II) oxidizing aerobic acidophiles (Elbehti *et al*., 2000).

Although the *pioABC* operon is conserved in four sequenced *R. palustris* strains, only *R. palustris* TIE-1 has been rigorously tested for its ability to perform photoferrotrophy (Jiao and Newman, 2007). Comparison of this locus showed that *R. palustris* TIE-1 and *R. palustris* CGA009 are most closely related in locus organization and open reading frame (ORF) amino acid sequence identity, while *R. palustris* BisB18 and *R. palustris* BisA53 have variable locus organization and lower ORF amino acid sequence identity compared with their homologues in *R. palustris* TIE-1 (Fig. S1). Haem staining of the PioA protein showed that it was most abundant during photoferrotrophic growth but was also detected at lower levels during photoautotrophic growth on hydrogen (H_2_) (Jiao and Newman, 2007). This indicated that expression of this operon might be highly regulated. Recent microarray analysis performed on *R. palustris* CGA009 showed that *pioABC* expression decreased in a *δfixK* regulator mutant versus wild-type (WT) during microaerobic chemoheterotrophic growth on succinate (Rey and Harwood, 2010). Although these results implied that FixK might control expression of the *pioABC* operon in *R. palustris* TIE-1, whether this occurs under anoxic phototrophic conditions including photoferrotrophic growth was not tested.

FixK belongs to the CRP/FNR family of regulators, which is distinct from the Fur/Irr family of regulators traditionally known to control iron metabolic genes in a number of bacteria (Escolar *et al*., 1999; Hantke, 2001; Johnston *et al*., 2007). FixK was first identified in *Sinorhizobium meliloti* (Batut *et al*., 1989). It binds a palindromic sequence 5'-TTGA(N6)TCAA-3', which has been dubbed the FixK box (Green *et al*., 1996; Nellen-Anthamatten *et al*., 1998). FixK is part of the FixLJ two-component regulatory system that has been well characterized in rhizobial species such as *Bradyrhizobium japonicum* and *Sinorhizobium meliloti*, as well as the non-rhizobial species *Caulobacter crescentus*. This regulatory system allows these organisms to sense low oxygen (O_2_) and change the expression of numerous genes permitting adaptation to microoxic as well as anoxic conditions (Crosson *et al*., 2005; Bobik *et al*., 2006; Mesa *et al*., 2008). FixL and FixJ act as a classical histidine kinase–reponse regulator pair leading to activation of *fixK* under low O_2_. FixK then relays this signal by modulating global gene expression (Gilles-Gonzalez and Gonzalez, 2005). Although FixK homologues exist in all rhizobial species known, they are not found in purple non-sulphur bacteria other than *R. palustris* (Cosseau and Batut, 2004; Rey and Harwood, 2010).

This study was initiated to understand the expression pattern of the *pioABC* operon and determine the regulatory mechanism that controls its expression in the photoferrotroph *R. palustris* TIE-1. By using both genetic and biochemical approaches, we identified FixK as an activator of the *pioABC* operon as well as other genes involved in regulation, photosynthesis, respiration and transport.

## Results

### *pioABC* expression is induced during anaerobic growth

**Figure 1:**
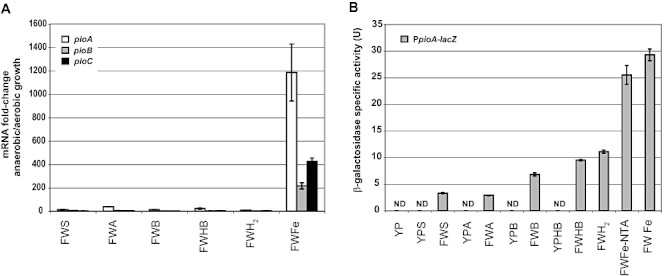
Expression of the *pioABC* operon was tested using two approaches. (A) qRT-PCR and (B) translational *lacZ* fusion to the *pioABC* promoter. FW, fresh water minimal medium for anaerobic phototrophic growth; YP, yeast- and peptone-rich medium for aerobic chemotrophic growth; S, succinate; A, acetate; B, benzoate; HB, 4-hydroxybenzoate; H_2_, hydrogen; Fe-NTA, FeCl_2_ with nitrilotriacetic acid; Fe, FeCl_2_ alone; ND, not detectable. A. Fold change was calculated with *clpX* as the internal control and by using the δδCt method for comparative expression analysis.B. β-Galactosidase activity was determined using a modified Miller assay and normalized with total protein to get specific activity [represented in U – Units (μmol min^−1^ mg protein^−1^)]. Assays were performed as three independent triplicates ± standard error.

To assess differences in the expression of the *pioABC* operon in *R. palustris* TIE-1, our first approach was to use quantitative reverse transcription PCR (qRT-PCR). Comparison of the mRNA abundance of *pioA, pioB* and *pioC* under various growth conditions revealed that expression was lowest during aerobic chemoheterotrophic growth. This condition was therefore used as a baseline to calculate the relative fold change in mRNA abundance. Expression of the *pioABC* transcripts was highly upregulated during photoferrotrophic growth relative to aerobic chemoheterotrophic growth (Fig. 1A). Interestingly, *pioABC* mRNA transcripts were in general higher during anaerobic phototrophic growth, although transcript levels were significantly higher under photoferrotrophic conditions.

To complement our qRT-PCR transcriptional data, we constructed an integrative *lacZ* reporter to determine the *in vivo* expression of the *pioABC* operon under various conditions. The *pioABC* genes form an operon as shown previously (Jiao and Newman, 2007). With the assumption that a single promoter drives the expression of the *pioABC* operon, we fused the entire intergenic region between *pioA* and the upstream gene Rpal_0818, in frame with the ATG start codon of the *lacZ* gene of *Escherichia coli* that encodes β-galactosidase. This led to formation of a *lacZ* translational fusion that was subsequently integrated onto the chromosome of WT *R. palustris* TIE-1. The level of β-galactosidase activity measured from this strain represented a combination of transcription and translation initiation. Assessment of β-galactosidase activity under various growth conditions revealed that there was no detectable activity of the *pioABC* operon during aerobic growth. β-Galactosidase activity was observed under all anaerobic growth conditions tested, being highest during photoferrotrophic growth (Fig. 1B). β-Galactosidase activity was 10-fold higher during photoferrotrophic growth compared with photoheterotrophic growth on succinate. Overall, these data are qualitatively similar to what we observed with qRT-PCR.

### The *pioABC* transcription start site is a guanine residue upstream of which lie two canonical FixK binding sites

**Figure 2:**
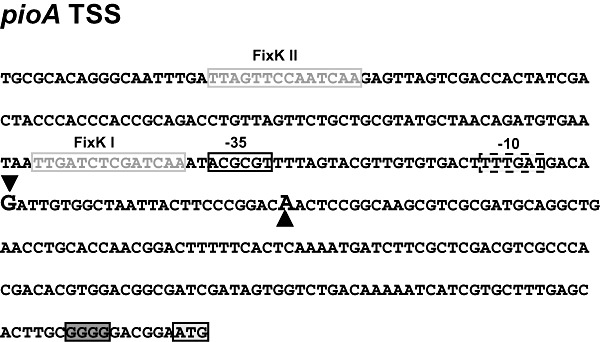
The transcription start site (TSS) of the *pioABC* operon was identified using cRACE and the processed site was identified using 5' RLM-RACE. The TSS was 177 bp upstream of the ATG (solid light grey box) start codon of *pioA* (upward black arrow). A processed site was observed 152 bp upstream of the ATG start codon of *pioA* (downward black arrow). A canonical FixK binding site is present 37 bp upstream of the TSS (FixK I) denoted by grey bases (lined grey box). Another potential FixK binding site is present 129 bp upstream of the TSS (FixK II) denoted by grey bases (lined grey box). The RBS is shown in solid dark grey box. The −10 box is denoted by a dashed line box and the −35 is denoted by a black box.

The transcription start site (TSS) of the *pioABC* operon was determined during photoheterotrophic growth on succinate, photoautotrophic growth on H_2_ as well as photoferrotrophic growth on Fe(II). cRACE and 5' RLM-RACE revealed that the TSS of the *pioABC* operon is a guanine residue 177 bp upstream of the predicted ATG start codon of *pioA* under all the growth conditions tested (Fig. 2 and Fig. S2). 5' RLM-RACE also revealed the presence of a processed site, which is an adenine residue 152 bp upstream of the predicted start codon. This processed site might be important for regulation mediated either by regulatory proteins or other mechanisms. A similar site has been observed in the *E. coli yfiD* promoter under the control of the Fnr protein (Green *et al*., 1998). Assessment of the region near the TSS revealed the presence of −10 and −35 core promoter elements of which only the −10 resembles the *E. coli*σf^70^ consensus (McClure, 1985). Centred at −44.5 is a canonical FixK box (named FixK I) assigning this promoter to a CRP/FNR class II promoter (Mesa *et al*., 2005). Another canonical FixK box (FixK II) is centred at position −137.5. Use of two identical binding sites has been observed for interaction of Fnr with the *yfiD* promoter and CRP interaction with the *acsP2* promoter in *E. coli* (Green and Baldwin, 1997; Beatty *et al*., 2003).

### Both identified FixK binding sites influence expression of the *pioABC* operon *in vivo*

**Figure 3:**
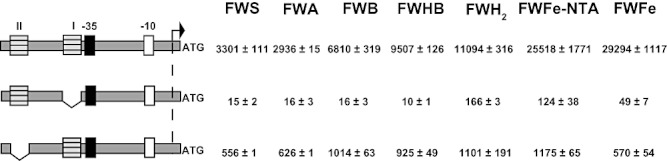
β-Galactosidase activity was determined for translational fusions with FixK I and FixK II boxes deleted. FW, fresh water minimal medium for anaerobic phototrophic growth; S, succinate; A, acetate; B, benzoate; HB, 4-hydroxybenzoate; H_2_, hydrogen; Fe-NTA, FeCl_2_ with nitrilotriacetic acid; Fe, FeCl_2_ alone. The grey-hashed boxes represent the FixK I and II boxes. The black box represents the −35 and the white box represents the −10. The black bent arrow represents the TSS. β-Galactosidase activity was determined using a modified Miller assay and normalized with total protein to get specific activity [milliUnits-mU (nmol min^−1^ mg protein^−1^)]. Values represent average of three independent triplicates ± standard error.

To assess the importance of the canonical FixK binding sites conserved in the *pioABC* promoter, we constructed DNA templates lacking either FixK I or FixK II boxes and fused them in frame with *lacZ*, forming translational fusions. These constructs were then integrated onto the chromosome of WT *R. palustris* TIE-1 and assayed for β-galactosidase activity on various growth substrates. Deletion of FixK I led to dramatic downregulation of β-galactosidase activity on all the substrates tested (Fig. 3). These data demonstrated that FixK I was important for activation of expression of the *pioABC* operon. Only a modest downregulation of β-galactosidase activity was observed when FixK II was deleted. This suggests that although the primary DNA site that influences *pioABC* expression is FixK I, FixK II does play a role in activating *pioABC* expression.

### *δfixK* has a phototrophic iron oxidation defect and is severely impaired during anaerobic phototrophic growth

**Figure 4:**
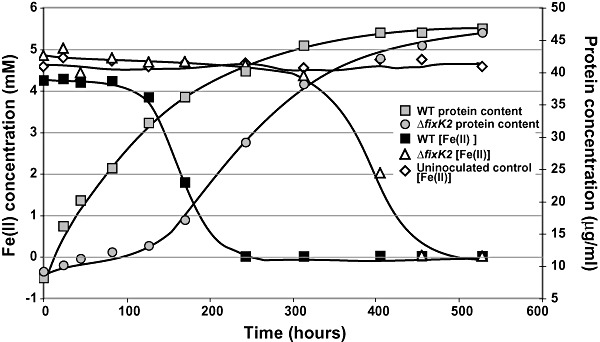
Deletion of *fixK* affects the ability of *R. palustris* TIE-1 to oxidize Fe(II). WT and δ*fixK* were pre-grown on hydrogen and inoculated into FW medium with Fe(II). The soluble Fe(II) concentration was monitored using the Ferrozine assay. Growth was monitored as increase in protein content as described in *Experimental procedures*. This experiment was performed thrice and one representative experiment is shown.

The identification of FixK binding sites in the *pioABC* promoter and the influence of these sites on *pioABC* expression implicated the FixK protein in controlling expression of this operon. We thus deleted this gene (Rpal_4729) from the chromosome of the P*pioA–lacZ* translational fusion strain and confirmed the mutant using PCR (Fig. S3). This *δfixK* strain was then characterized with respect to phototrophic Fe(II) oxidation and defects in growth (Fig.4 and Table S1). Comparison of the ability of *δfixK* and WT *R. palustris* TIE-1 to oxidize Fe(II) phototrophically revealed that *δfixK* was severely delayed in photoferrotrophic growth (Fig. 4). WT *R. palustris* TIE-1 started oxidizing Fe(II)-NTA at ˜120 h after inoculation while *δfixK* showed first signs of Fe(II)-NTA oxidation at ˜320 h post inoculation. Eventually, both WT and *δfixK* were able to oxidize 5 mM Fe(II) with 10 mM NTA to completion as well as achieve similar levels of total protein content. The rate of Fe(II)-NTA oxidation by WT and *δfixK* also appeared to be similar [˜0.03 mM Fe(II)-NTA oxidized per hour]. No significant difference in lag time was observed between WT and *δfixK* during aerobic growth on YP medium, although slight differences in generation time were detected (Table S1). In contrast, a substantial increase in lag time was observed in *δfixK* versus WT during anaerobic phototrophic growth in FW medium. In general, the *δfixK* mutant had lower pigmentation both during aerobic chemoheterotrophic growth as well as phototrophic growth (Fig. S4). A similar defect was observed in *R. palustris* CGA009 and was attributed to production of lower amounts of reaction centre as well as light harvesting complexes 1 and 2 (LH1 and 2) (Rey and Harwood, 2010). Moreover, the expression of genes for the LH1 complex (*pufAB*) was found to decrease slightly in the *δfixK* mutant (Rey and Harwood, 2010). Reasoning that the lower pigmentation in the *R. palustris* TIE-1 *δfixK* mutant was likely due to similar expression changes, we next sought to determine whether the phototrophic Fe(II) oxidation defect was an indirect effect or due to a direct interaction between the *pioABC* promoter with FixK.

### FixK affects expression of the *pioABC* operon

**Figure 5:**
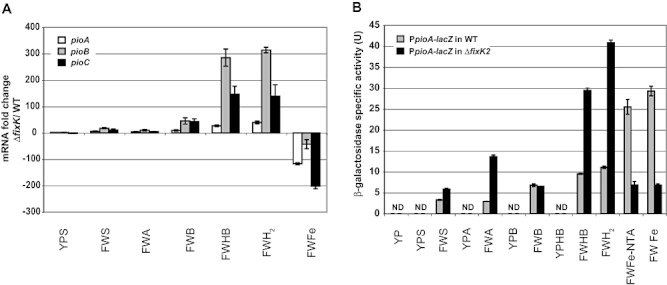
Comparison of *pioABC* expression between δ*fixK* and WT reveals that FixK affects this operon's expression. (A) qRT-PCR and (B) translational *lacZ* fusion to the *pioABC* promoter. FW, fresh water minimal medium for anaerobic phototrophic growth; YP, yeast- and peptone-rich medium for aerobic chemotrophic growth; S, succinate; A, acetate; B, benzoate; HB, 4-hydroxybenzoate; H_2_, hydrogen; Fe-NTA, FeCl_2_ with nitrilotriacetic acid; Fe, FeCl_2_ alone; ND, not detectable. A. Fold change was calculated with *clpX* as the internal control and by using the δδCt method for comparative expression analysis. B. β-Galactosidase activity was determined using a modified Miller assay and normalized with total protein to get specific activity [represented in U – Units (μmol min^−1^ mg protein^−1^)]. Values represent average of three independent triplicates ± standard error.

To assess whether FixK directly affects *pioABC* expression, we determined expression of this operon in *δfixK* versus WT using qRT-PCR and translational reporter gene fusions (Fig. 5). No significant difference was observed between *pioABC* transcripts levels in the *δfixK* mutant versus WT during aerobic chemoheterotrophic growth on succinate (Fig. 5A). However, expression of the *pioABC* genes increased in the *δfixK* mutant versus WT under all the phototrophic conditions tested except during photoferrotrophic growth, when *pioABC* mRNA decreased in the mutant. The translational fusion data coincided with the mRNA abundance data and showed that β-galactosidase activity increased in the *δfixK* mutant versus WT under most phototrophic conditions tested but decreased during photoferrotrophic growth. The decrease in β-galactosidase activity during photoferrotrophic growth was approximately fivefold. The stability of the β-galactosidase enzyme might account for the modest decrease in expression from the P*pio–lacZ* fusion compared with the dramatic decrease in *pioABC* mRNA abundance. Overall, these data suggest that FixK either directly or indirectly regulates expression of the *pioABC* operon in response to growth conditions. The only condition we identified under which FixK activated *pioABC* expression was photoferrotrophy, whereas repression occurred on all other phototrophic conditions tested. Complementation of the *δfixK* mutant with either WT or N-terminal His_6_-tagged FixK resulted in partial restoration of *pioABC* expression as well as full restoration of FixK expression during photoferrotrophic growth (Fig. S4). The complemented strains had similar pigmentation to WT during phototrophic growth most likely due to restored production of haem, bacteriochlorophyll and LH1 and LH2 complex proteins similar to that observed in *R. palustris* CGA009 (Fig. S4) (Rey and Harwood, 2010). Why only partial restoration of *pioABC* expression occurred is unclear.

### FixK directly binds the FixK I box of the *pioABC* promoter

**Figure 6:**
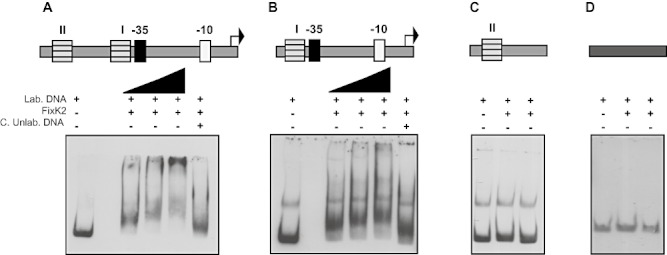
Gel mobility-shift assays were performed using purified Nterm-His_6_-FixK protein. A. A double-stranded DNA substrate encompassing both FixK I and II binding sites was tested for the ability to bind FixK. B. A double-stranded DNA substrate encompassing only the FixK I binding sites was tested for the ability to bind FixK. C. A double-stranded DNA substrate encompassing only the FixK II binding site was tested for the ability to bind FixK. D. A double-stranded DNA substrate encompassing only the Oct2A binding site was tested for the ability to bind FixK. Approximately 30 fmol of labelled DNA (Lab. DNA) was used with twofold increase in purified FixK protein starting from 2.9 μM (represented by the black triangle). A 100-fold higher concentration of competitive unlabelled DNA (C. Unlab. DNA) was added to determine specificity of binding when 8.7 μM FixK was added to the reaction.

To determine whether FixK could directly interact with the identified FixK I and FixK II boxes, we performed gel mobility-shift assays using recombinant FixK protein (Fig. S5 and Fig. 6). These experiments showed that purified FixK protein was able to bind a double-stranded (ds) DNA substrate that had the FixK I box as well as a dsDNA substrate that had both the FixK I and FixK II box. However, purified FixK did not bind a dsDNA substrate that had only the FixK II box. FixK binding was specific as it could be competed out with competitive unlabelled DNA. In addition, purified FixK was unable to bind a non-cognate, Oct2A (a eukaryotic transcription factor; Corcoran and Shore, 2000) binding site containing dsDNA substrate (provided in the DIG Gel Shift Kit, Second Generation; Roche, Indianapolis, IN). Overall, these data suggest that FixK binds the *pioABC* promoter at the FixK I box but not the FixK II box, thereby mediating regulation of this operon. Although the FixK II box was observed to be important for expression *in vivo*, it did not bind FixK. This implies that either it binds other proteins in the CRP/FNR family or can bind FixK only under specific conditions absent in our *in vitro* assay. DNA topology of the FixK II box and/or the low binding affinity of FixK protein for this site might also account for this result.

### Putative FixK target genes are upregulated during anaerobic phototrophic growth

The *R. palustris* CGA009 genome was scanned for the presence of potential FixK binding sites in a previous study (Conlan *et al*., 2005). Canonical FixK boxes were observed in the promoter regions of 21 ORFs in this organism including *pioA*. We therefore identified homologues of these genes in *R. palustris* TIE-1 and assessed the change in their expression using qRT-PCR. The fold change in mRNA abundance was calculated with respect to aerobic chemoheterotrophic growth on succinate (Table 1). This revealed that a number of selected genes are upregulated more than fivefold during phototrophic growth. These genes encompassed a number of different aspects of the physiology of *R. palustris* TIE-1 including respiration, photosynthesis, gene regulation and transport. A detailed description of these genes can be found in the supporting information. Their upregulation during anaerobic growth suggests that they are likely part of the FixK regulon in *R. palustris* TIE-1 that allows it to adapt to anoxia as shown recently for *R. palustris* CGA009 under microoxic conditions (Rey and Harwood, 2010).

**Table 1:** Fold change^a^ in mRNA abundance of genes likely controlled by FixK during anaerobic versus aerobic growth on various substrates.

Locus tag	Gene annotation and likely function	Fold change during anaerobic versus aerobic growth
Succinate	Acetate	Benzoate	4-hydroxy benzoate	Hydrogen	Fe(II)
Regulation							
Rpal_1207	Transcriptional regulator, PadR-like family	3.7 ± 0.5	2.9 ± 0.3	3.9 ± 0.6	4.6 ± 0.1	3.9 ± 1.1	5.1 ± 0.6
Rpal_1280	CRP/FNR family protein	7.9 ± 1.0	12.7 ± 1.7	4.4 ± 0.4	8.5 ± 1.3	2.5 ± 0.7	40.4 ± 3.8
Rpal_2583	Ferric-uptake regulator (possibly Irr)	91.8 ± 16.5	73.3 ± 14.4	21.9 ± 5.1	54.7 ± 11.8	70.5 ± 5.0	13.0 ± 1.3
Rpal_4713	Anaerobic aromatic degradation regulator AadR, CRP/FNR family (*aadR*)	6.7 ± 1.1	5.4 ± 1.8	4.5 ± 1.3	4.0 ± 0.7	2.6 ± 0.2	10.1 ± 3.2
Rpal_4729	FixK (*fixK*)	8.7 ± 2.0	2.4 ± 0.4	14.0 ± 3.3	38.5 ± 11.7	9.8 ± 2.4	12.2 ± 2.4
Photosynthesis							
Rpal_0922	Haem and sirohaem biosynthesis protein HemO (*hemO*)	6.5 ± 0.7	10.5 ± 1.5	5.3 ± 0.4	5.8 ± 0.6	4.8 ± 1.1	14.3 ± 0.2
Rpal_1692	Porphobilinogen deaminase (*bchD*)	9.9 ± 1.6	28.2 ± 1.3	17.4 ± 2.7	17.1 ± 3.2	21.9 ± 7.7	16.1 ± 0.1
Rpal_2130	Cytochrome C biogenesis protein (*cycH*)	6.2 ± 0.2	8.3 ± 2.0	5.4 ± 0.3	11.1 ± 1.0	3.0 ± 0.8	23.2 ± 5.1
Respiration							
Rpal_0020	Cytochrome oxidase CcoN(OQP)(*ccoN*)	1.3 ± 0.1	5.2 ± 1.4	2.7 ± 0.8	2.7 ± 0.6	2.3 ± 0.1	2.7 ± 0.1
Rpal_1206	Gene next to *ccoG*	2.2 ± 0.4	2.4 ± 0.5	2.1 ± 0.8	3.7 ± 0.2	1.4 ± 0.3	2.2 ± 0.5
Transport							
Rpal_1412	Hypothetical protein with signal peptide and transmembrane domains	9.3 ± 0.2	26.6 ± 5.3	22.8 ± 1.8	34.7 ± 8.2	18.0 ± 3.4	11.2 ± 0.1
Rpal_1868	Transport-associated and nodulation region associated ORF (*osmY*)	18.6 ± 2.3	10.0 ± 0.2	3.1 ± 0.6	2.3 ± 0.4	3.3 ± 0.2	6.4 ± 1.8
Rpal_2582	Predicted ORF in operon with heavy metal transporter	246.0 ± 64.8	264.8 ± 33.0	68.7 ± 14.6	127.2 ± 5.1	77.7 ± 14.9	52.7 ± 14.3
Rpal_3436	Putative potassium uptake protein Kup1 (*kup1*)	3.6 ± 0.3	4.4 ± 0.9	1.9 ± 0.1	1.8 ± 0.4	3.5 ± 0.8	4.3 ± 0.1
Rpal_4015	Uncharacterized protein involved in siderophore biosynthesis	23.9 ± 1.7	76.3 ± 14.2	36.9 ± 6.1	45.2 ± 0.2	30.5 ± 8.2	81.7 ± 2.4
Rpal_4717	Putative ABC transporter	0.4 ± 0.1 (3.0 ± 0.9)	1.1 ± 0.2	0.4 ± 0.1 (2.5 ± 0.5)	0.4 ± 0.1 (2.8 ± 1.0)	0.6 ± 0.1 (1.8 ± 0.2)	1.5 ± 0.2
Rpal_4994	Putative outer membrane protein (*ompW*)	15.8 ± 1.6	83.4 ± 10.1	19.8 ± 3.7	27.0 ± 2.2	31.6 ± 5.0	2.8 ± 0.4
Other functions							
Rpal_1413	Inosine-5'-monophosphate dehydrogenase	1.5 ± 0.4	4.8 ± 0.1	2.1 ± 0.7	1.8 ± 0.6	0.7 ± 0.1 (1.2 ± 0.3)	2.1 ± 0.3
Rpal_1691	Hypothetical protein	5.4 ± 0.3	7.8 ± 0.2	5.3 ± 1.4	5.3 ± 0.3	2.2 ± 0.1	12.2 ± 1.4
Rpal_1869	Putative phosphoketolase	2.3 ± 0.5	2.4 ± 0.8	1.6 ± 0.1	1.4 ± 0.3	0.7 ± 0.1 (1.2 ± 0.3)	10.1 ± 1.2
Rpal_2453	Putative short chain dehydrogenase	2.3 ± 0.6	4.0 ± 1.5	3.7 ± 0.9	4.3 ± 0.8	1.8 ± 0.3	3.1 ± 0.4

**a.** Fold change was calculated as indicated in *Experimental procedures*. Numbers > 1 represent higher mRNA abundance under anaerobic versus aerobic growth. Numbers < 1 represent lower mRNA abundance under anaerobic versus aerobic growth. The values in parentheses represent this decrease in mRNA abundance. Values represent the average of three independent cultures assayed in triplicate ± the standard error.

### FixK affects the expression of a number of predicted target genes

To establish the influence of FixK on expression of the putative target genes, we determined their level of expression in WT *R. palustris* TIE-1 using qRT-PCR and compared it with *δfixK* (Table 2). Only one gene, encoding a putative potassium uptake protein (*kup1*), showed more than fivefold increase in expression in *δfixK* under anaerobic growth conditions (Schleyer and Bakker, 1993). Transcripts for this gene were not significantly upregulated during any anaerobic growth condition in the WT (Table 1). In contrast, expression of 14 of the 20 putative FixK target genes assessed decreased in *δfixK*. *hemO*, *ccoN(OQP)* and *bchD*, all photosynthetic genes, showed a dramatic decrease in *δfixK* during phototrophic growth. The regulatory genes selected were also downregulated more than fivefold on at least one anaerobic growth condition in *δfixK*. Some other interesting genes encoding transport-related functions also emerged as FixK targets such as Rpal_4015 (predicted ORF involved in siderophore biosynthesis), Rpal_4994 (*ompW* homologue), Rpal_1868 (*osmY* homologue), Rpal_2582 (first gene of a heavy metal transporting operon) and Rpal_1412 (a signal peptide containing ORF with transmembrane domains). Two ORFs Rpal_1413 (putative inosine-5'-monophosphate dehydrogenase) (Zhang *et al*., 1999) and Rpal_1691 (hypothetical protein) were also modestly downregulated in *δfixK*.

**Table 2:** Fold change^a^ in mRNA abundance of genes likely controlled by FixK in the *δfixK* mutant versus WT on various growth substrates.

Locus tag	Gene annotation and likely function	Fold change in *δfixK* mutant versus WT
Succinate	Acetate	Benzoate	4-hydroxy benzoate	Hydrogen	Fe(II)
Regulation							
Rpal_1207	Transcriptional regulator, PadR-like family	0.1 ± 0.0.01 (8.4 ± 0.7)	1.1 ± 0.4	0.17 ± 0.01 (5.4 ± 0.1)	0.43 ± 0.07 (2.4 ± 0.6)	0.18 ± 0.05 (5.7 ± 1.6)	0.7 ± 0.2 (1.3 ± 0.1)
Rpal_1280	CRP/FNR family protein	0.1 ± 0.03 (9.2 ± 3.0)	0.3 ± 0.06 (3.8 ± 0.9)	0.14 ± 0.02 (7.2 ± 1.6)	0.4 ± 0.1 (2.9 ± 0.7)	0.16 ± 0.04 (6.2 ± 1.7)	0.13 ± 0.01 (7.9 ± 1.1)
Rpal_2583	Ferric-uptake regulator (possibly Irr)	0.04 ± 0.01 (26.6 ± 4.5)	0.2 ± 0.04 (5.0 ± 1.2)	0.5 ± 0.2 (2.8 ± 1.6)	4.3 ± 0.8	0.12 ± 0.03 (8.93 ± 2.9)	1.49 ± 0.01
Rpal_4713	Anaerobic aromatic degradation regulator AadR, CRP/FNR family (*aadR*)	0.2 ± 0.05 (4.2 ± 0.8)	0.2 ± 0.08 (7.7 ± 0.3)	0.2 ± 0.01 (6.0 ± 0.4)	0.64 ± 0.04 (1.6 ± 0.1)	0.7 ± 0.2 (1.6 ± 0.4)	0.34 ± 0.03 (3.0 ± 0.3)
Rpal_4729	FixK (*fixK*)	ND	ND	ND	ND	ND	ND
Photosynthesis							
Rpal_0922	Haem and sirohaem biosynthesis protein HemO (*hemO*)	0.2 ± 0.004 (5.2 ± 0.1)	0.1 ± 0.01 (7.9 ± 0.3)	0.3 ± 0.1 (3.7 ± 1.4)	1.5 ± 0.2	0.12 ± 0.01 (8.3 ± 0.4)	0.02 ± 0.002 (57.5 ± 9.2)
Rpal_1692	Porphobilinogen deaminase (*bchD*)	0.03 ± 0.01 (31.6 ± 6.2)	0.02 ± 0.003 (40.6 ± 4.9)	0.09 ± 0.02 (10.3 ± 2.0)	0.09 ± 0.02 (10.8 ± 2.3)	0.05 ± 0.01 (17.7 ± 3.4)	0.01 ± 0.002 (85.6 ± 15.3)
Rpal_2130	Cytochrome C biogenesis protein (*cycH*)	0.25 ± 0.01 (4.0 ± 0.1)	0.4 ± 0.1 (2.5 ± 0.6)	0.4 ± 0.2 (2.7 ± 1.2)	0.50 ± 0.07 (2.2 ± 0.3)	0.38 ± 0.05 (2.6 ± 0.3)	0.3 ± 0.1 (3.4 ± 1.6)
Respiration							
Rpal_0020	Cytochrome oxidase CcoN(OQP)(*ccoN*)	0.07 ± 0.02 (13.8 ± 3.1)	0.04 ± 0.006 (24.7 ± 3.5)	0.15 ± 0.02 (6.7 ± 0.9)	0.10 ± 0.01 (9.3 ± 1.0)	0.06 ± 0.01 (15.9 ± 0.1)	0.008 ± 0.001 (129.1 ± 11.2)
Rpal_1206	Gene next to *ccoG*	0.14 ± 0.03 (7.5 ± 1.9)	0.4 ± 0.1 (2.4 ± 0.8)	0.3 ± 0.03 (4.0 ± 0.5)	0.14 ± 0.03 (7.0 ± 1.9)	0.25 ± 0.01 (4.0 ± 0.2)	0.21 ± 0.04 (4.8 ± 0.9)
Transport							
Rpal_1412	Hypothetical protein with signal peptide and transmembrane domains	0.08 ± 0.004 (13.3 ± 0.7)	0.03 ± 0.003 (31.7 ± 3.3)	0.09 ± 0.02 (10.3 ± 2.0)	0.30 ± ± 0.05 (3.3 ± 0.6)	0.09 ± 0.01 (10.4 ± 0.9)	0.04 ± 0.01 (30.2 ± 11.9)
Rpal_1868	Transport-associated and nodulation region associated ORF (*osmY*)	0.01 ± 0.002 (145.1 ± 48)	0.03 ± 0.001 (39.5 ± 2.1)	0.06 ± 0.01 (18.7 ± 4.6)	0.4 ± 0.1 (2.8 ± 0.7)	0.03 ± 0.01 (26.3 ± 2.7)	0.08 ± 0.01 (12.4 ± 1.4)
Rpal_2582	Predicted ORF in operon with heavy metal transporter	0.03 ± 0.01 (34.3 ± 7.7)	0.04 ± 0.005 (22.4 ± 2.5)	0.41 ± 0.03 (2.4 ± 0.2)	1.4 ± 0.1	0.25 ± 0.04 (3.9 ± 0.6)	0.10 ± 0.02 (9.4 ± 1.9)
Rpal_3436	Putative potassium uptake protein Kup1 (*kup1*)	0.2 ± 0.05 (5.1 ± 1.3)	8.2 ± 1.8	1.0 ± 0.1	5.3 ± 1.0	2.1 ± 0.8	0.22 ± 0.01 (4.6 ± 0.1)
Rpal_4015	Uncharacterized protein involved in siderophore biosynthesis	0.08 ± 0.003 (13.1 ± 0.6)	0.03 ± 0.002 (26.4 ± 2.1)	0.11 ± 0.02 (9.3 ± 1.8)	0.24 ± 0.04 (4.2 ± 0.6)	0.08 ± 0.01 (11.7 ± 1.7)	0.2 ± 0.01 (4.9 ± 0.2)
Rpal_4717	Putative ABC transporter	0.8 ± 0.2 (1.2 ± 0.3)	1.0 ± 0.1	0.7 ± 0.2 (1.5 ± 0.6)	1.8 ± 0.4	0.7 ± 0.1 (1.4 ± 0.3)	0.36 ± 0.1 (2.8 ± 0.6)
Rpal_4994	Putative outer membrane protein (*ompW*)	0.04 ± 0.01 (25.4 ± 5.1)	0.02 ± 0.001 (59.1 ± 3.9)	0.40 ± 0.04 (2.7 ± 0.3)	0.33 ± 0.01 (3.1 ± 0.1)	0.17 ± 0.06 (6.21 ± 2.2)	0.15 ± 0.01 (6.6 ± 0.5)
Other functions							
Rpal_1413	Inosine-5'-monophosphate dehydrogenase	0.2 ± 0.04 (5.0 ± 0.9)	0.1 ± 0.01 (7.4 ± 0.7)	0.7 ± 0.1 (1.5 ± 0.2)	1.6 ± 0.3	0.9 ± 0.3 (1.3 ± 0.5)	0.09 ± 0.04 (11.6 ± 3.6)
Rpal_1691	Hypothetical protein	0.3 ± 0.03 (3.3 ± 0.4)	0.3 ± 0.06 (4.2 ± 1.2)	0.4 ± 0.1 (2.3 ± 0.6)	0.6 ± 0.2 (1.9 ± 0.1)	0.5 ± 0.1 (2.2 ± 0.7)	0.04 ± 0.01 (25.0 ± 3.2)
Rpal_1869	Putative phosphoketolase	0.6 ± 0.3 (2.0 ± 0.8)	1.1 ± 0.5	0.7 ± 0.1 (1.4 ± 0.2)	1.5 ± 0.5	2.1 ± 0.9	0.11 ± 0.02 (9.4 ± 1.9)
Rpal_2453	Putative short chain dehydrogenase	0.2 ± 0.05 (4.9 ± 1.0)	1.1 ± 0.3	0.3 ± 0.04 (3.6 ± 0.7)	0.54 ± 0.07 (1.9 ± 0.2)	0.45 ± 0.07 (2.3 ± 0.4)	5.0 ± 0.05

a. Fold change was calculated as indicated in *Experimental procedures*. Numbers > 1 represent higher mRNA abundance in *δfixK* versus WT. Numbers < 1 represent lower mRNA abundance in *δfixK* versus WT. The values in parentheses represent decrease in mRNA abundance. ND, not detectable. Values represent the average of three independent cultures assayed in triplicate ± the standard error.

## Discussion

In this study, we showed that the phototrophic iron oxidation genes of *R. palustris* TIE-1 were expressed under all anaerobic phototrophic growth conditions tested. Interestingly, expression was further induced during photoferrotrophic growth. FixK regulates *pioABC* expression, although its mode of regulation varies based on the growth condition. Although Fur family proteins might also contribute to *pioABC* expression, the involvement of FixK indicates that the regulation of iron metabolism in this organism lies outside the canonical Fur/Irr paradigm. We identified FixK target genes under anaerobic phototrophic growth, some of which are homologues of those identified as part of the FixK regulon under microoxic conditions in *R. palustris* CGA009 (Rey and Harwood, 2010). Additional novel FixK targets were also revealed. This study represents the first expression and regulation analysis of photoferrotrophic genes in any organism, and raises a number of physiological and mechanistic questions.

### Why are *pio* genes induced by anoxia?

The *pioABC* operon was shown to be essential for photoferrotrophic growth by *R. palustris* TIE-1 (Jiao and Newman, 2007). The deletion of this operon had no effect on growth under other growth conditions (Jiao and Newman, 2007). Yet we observed that it was transcribed and translated under all anaerobic phototrophic conditions. This was unexpected and pointed to the possibility that the Pio proteins might serve a function other than supporting photoferrotrophic growth. In this regard, it is interesting to note that it was recently observed that phototrophic Fe(II) oxidation might serve as a detoxification mechanism for *Rhodobacter capsulatus* strain SB1003 in the presence of low micromolar concentrations of Fe(II) (Poulain and Newman, 2009). If PioABC were to serve a similar function for *R. palustris* TIE-1, then we could rationalize expression of this operon during anaerobic growth even in the absence of high levels of Fe(II). An alternative explanation is that even though the *pio* genes are transcribed and translated, the fully functional decahaem cytochrome PioA does not form except during photoferrotrophic growth due to lack of sufficient or appropriate maturation proteins. Because haem-containing cytochromes such as PioA require additional maturation, such a situation is conceivable and is in agreement with the haem staining data reported previously (Stevens *et al*., 2005; Jiao and Newman, 2007). Whether apo-PioA has a biological function independent of Fe(II) oxidation remains to be determined.

### How does FixK regulate *pioABC* expression?

The mode of regulation of this operon is most likely via activation by FixK. This is based on the location of the FixK I binding site that interacts directly with the purified protein *in vitro* at −44.5 (Mesa *et al*., 2005). This promoter resembles a class II CRP/FNR promoter, where it is expected to make contacts with domain 4 of the sigma factor thus activating transcription (Browning and Busby, 2004). Mutant analysis combined with the *in vitro* data suggests that this activation happens specifically during photoferrotrophic growth. Moreover, the deletion of the FixK I and FixK II binding sites leads to a drastic downregulation of *pioABC* expression, supporting an activator function of FixK. Intriguingly, expression of the *pioABC* operon increased in *δfixK* during non-photoferrotrophic growth, suggesting that FixK acts as a repressor of *pioABC* expression on other growth substrates. Based on the binding site results, this effect is likely indirect. Future studies using *in vitro* transcription assays might confirm the direct activation role of FixK. Additional levels of *pioABC* regulation clearly exist and await discovery.

### How similar is the *R. palustris* CGA009 microoxic chemoheterotrophic response to the *R. palustris* TIE-1 anoxic phototrophic response?

In a recent study, the role of FixK in the microoxic chemoheterotrophic response of *R. palustris* CGA009 was reported (Rey and Harwood, 2010). This study showed that the FixK regulon included genes for microaerobic respiration, phototrophy, autotrophy and aromatic compound degradation. This data set also revealed that during microaerobic chemotrophic growth on succinate, the *pioABC* genes were expressed and positively regulated by FixK. Although it has never been shown whether *R. palustris* CGA009 can perform photoferrotrophy, the *pioABC* genes were clearly expressed in this organism under microoxic non-photoferrotrophic conditions. Our data on *R. palustris* TIE-1 showed that there was no difference in *pioABC* mRNA abundance between WT and *δfixK* during aerobic chemotrophic growth. In contrast to what was observed during microaerobic growth in *R. palustris* CGA009, during anaerobic growth in *R. palustris* TIE-1, expression of the *pioABC* operon increased in *δfixK* under non-photoferrotrophic conditions. Therefore, a disparity exists in *pioABC* expression data, which might be due to the use of microoxic conditions for *R. palustris* CGA009 growth versus anoxic conditions for *R. palustris* TIE-1. Comparison of expression of other FixK targets between the two data sets showed similarities; expression of some common regulators decreased in *δfixK* along with genes encoding photosynthetic proteins and microaerobic respiratory proteins (Rey and Harwood, 2010). Some new targets emerged from our data set, most of which encode transport-related functions (Table 2).

In the coming years, it will be interesting to learn what other factors are necessary to support photoferrotrophic growth, or anaerobic growth in the presence of Fe(II) more generally, and what other additional layers of regulation beyond FixK are involved.

## Experimental procedures

### Bacterial strains, media and growth conditions

All strains used and constructed in this study are indicated in Table S2. *E. coli* strains were routinely grown in Luria–Bertani (LB) broth at 37°C with shaking at 250 r.p.m. For aerobic chemoheterotrophic growth, *Rhodopseudomonas palustris* TIE-1 was grown in 100 mM MOPS [3-N (morpholino) propanesulphonic acid] pH 7.0 0.3% Yeast extract and 0.3% Peptone (YP) medium in the dark at 30°C with shaking at 250 r.p.m. For anaerobic phototrophic growth *R. palustris* TIE-1 strains were grown in anoxic bicarbonate buffered freshwater (FW) medium (Jiao *et al*., 2005). For photoheterotrophic growth the FW medium was supplemented with anoxic 1 M stocks at pH 7.0 of sodium succinate, sodium acetate, sodium benzoate and sodium 4-hydroxybenzoate to a final concentration of 1 mM and incubated at 30°C in a Percival Intellus Environmental Controller Model – AR22LC8 fitted with two 60 W tungsten bulbs providing total irradiance of ˜40 W m^−2^. For photoautotrophic growth on H_2_, *R. palustris* TIE-1 was grown in FW medium pressurized with 50 kPascal of H_2_/CO_2_ (80%/20%). For photoautotrophic growth on Fe(II), FW medium was prepared under the flow of 34.5 kPascal N_2_/CO_2_ (80%/20%) and dispensed into sterile serum bottles/Balch tubes purged with 34.5 kPascal N_2_/CO_2_ (80%/20%). The container was then sealed using sterile butyl rubber stoppers and stored at room temperature for at least a day before supplementing with anoxic sterile stocks of FeCl_2_ to a final concentration of 5 mM and nitrilotriacetic (NTA) acid to a final concentration of 10 mM when required. For growth on solidified medium, LB or YP medium was solidified with 1.5% agar and supplemented with gentamicin at 20 μg ml^−1^ (*E. coli*) and 800 μg ml^−1^ (*R. palustris* TIE-1). For growth curve experiments and doubling time calculations, exponential phase cultures were inoculated into appropriate media at 10^−2^ dilution and optical density (OD) was monitored at 660 nM. The OD_660_ measurements were plotted versus time on a log scale and the slope of the curve was used to determine the growth constant *k*. The doubling time, *g*, was calculated from the following equation: *g* = ln(2)/*k*.

### DNA methods, plasmid and strain construction

All plasmid constructions and primers used in this study are indicated in Tables S2 and S3. All primers used in this study were obtained from Integrated DNA Technologies, Coralville, IA. A QIAprep Spin Miniprep kit (Qiagen, Valencia, CA, USA) was used for isolation of plasmid DNA from *E. coli*. Genomic DNA was isolated from *R. palustris* TIE-1 cells using the DNeasy Blood and Tissue kit (Qiagen, Valencia, CA, USA) and used as template for PCR reactions. All nucleic acids isolated in this study were quantified when necessary using a Nanodrop 1000 Spectrophotometer (Thermo Scientific, Waltham, MA). DNA sequencing was performed to confirm identity of all DNA constructs at the Biopolymers Laboratory in the Massachusetts Institute of Technology Center for Cancer Research. *E. coli* strains were transformed by electroporation using an Electroporator 2510 (Eppendorf, Hamburg, Germany), as recommended by the supplier. Plasmids were mobilized from *E. coli* S17-1/λpir into *R. palustris* TIE-1 by conjugation on YP agar plates as described previously (Jiao and Newman, 2007).

### Construction of a single integration system for *R. palustris* TIE-1

In order to employ *lacZ* reporter gene fusions in *R. palustris* TIE-1, it was imperative to ensure that the fusion was present in single copy on the chromosome of this organism. We designed an integrative system in *R. palustris* TIE-1 such that a desired region of DNA could be inserted onto the intergenic region of the operon Rpal_2933-2935 (*glmUS* homologue followed by an ORF of unknown function called gene *glmX* here) and Rpal_2936 (*recG* homologue). Details of the construction of this system can be found in the supporting information.

### Construction of a *lacZ* reporter system for *R. palustris* TIE-1

A pBBR1-based *lacZ* plasmid, pAB301 was designed for making translational fusions (Fig. S6). Details of this construct can be found in the supporting information. For assessing the expression of the *pioABC* operon of *R. palustris* TIE-1, the intergenic region between *pioABC* (Rpal_0817-0815) and Rpal_0818 (putative sulphate ABC transport subunit) was cloned into pAB301 forming pAB307. This entire cassette was re-amplified to incorporate NcoI sites on either side. This cassette was then cloned into the unique NcoI site of pAB314 (described in the supporting information) resulting in pAB322. This plasmid was used to insert the Ppio^TIE-1^−*lacZ* translational fusion onto the chromosome of WT *R. palustris* TIE-1, resulting in strain AB8.

### Construction of *R. palustris* TIE-1 *fixK* deletion mutant

The *δfixK* deletion mutant was constructed in *R. palustris* TIE-1 as described previously (Jiao and Newman, 2007). In short, the 1 kb upstream and 1 kb downstream region of the *fixK* ORF (Rpal_4729) was fused using overlap extension PCR as described previously (Bose and Metcalf, 2008). This PCR product was cloned into pJQ200KS resulting in pAB337. pAB337 was transferred to *R. palustris* TIE-1 strain AB8 using *E. coli* S17-1/λpir. The integration of the plasmid either at the *fixK* upstream or downstream region was selected by gentamicin resistance and the resulting integrants were screened by PCR. The integrants were grown selectively in the presence of gentamicin followed by two passages at 10^−2^ dilutions in non-selective YP medium. One integrant AB9 was chosen for segregation, which was achieved by plating on YP medium with 10% sucrose. Fifty sucrose resistant colonies were grown on plain YP medium and screened by PCR. One of the 50 colonies was a *fixK* (AB10) deletion mutant (as confirmed by PCR in Fig. S3) and was single colony purified 4 times on plain YP medium solidified with 1.5% agar.

### Complementation of the *δfixK* mutant

The *R. palustris* TIE-1 *fixK* gene was cloned such that an NdeI site was incorporated to overlap with the ATG start codon and a SpeI site was incorporated at the end of the gene. This PCR product was then cloned into pSRKGm giving rise to pAB363 (Khan *et al*., 2008). This plasmid allows controlled expression of the cloned gene driven by a modified Plac promoter. pAB363 was transferred to AB10 (*δfixK*) using the mating strain *E. coli* S17-1/λpir and selected on 800 μg ml^−1^ gentamicin. A single colony was chosen and grown on 800 μg ml^−1^ gentamicin with 10 mM IPTG (AB15). For subsequent purification of FixK protein an N-terminal 6× Histidine tag was chosen. To test whether this form of FixK was able to complement AB10 we cloned the N-terminal 6× Histidine tagged version of *fixK* into pSRKGm to give rise to pAB408. pAB408 was transferred to AB10 (*δfixK*) using mating strain *E. coli* S17-1/λpir and selected on 800 μg ml^−1^ gentamicin. A single colony was chosen and grown on 800 μg ml^−1^ gentamicin with 10 mM IPTG (AB20).

### Determination of transcription start sites

Transcription start sites were determined using two variations of rapid amplification of cDNA ends, namely, 5' RLM-RACE and cRACE (the primers used are indicated in Table S4). 5' RLM-RACE was performed as previously described with minor changes described in the supporting information (Bose and Metcalf, 2008). cRACE was performed as described previously with minor modifications described in the supporting information (Maruyama *et al*., 1995, Main-Hester *et al*., 2008; Rey and Harwood, 2010).

### Measurement of β-galactosidase activity

β-Galactosidase activity was measured by a variation of the method of Miller as described in the supporting information (Miller, 1992).

### Quantitative reverse-transcription PCR

For expression analysis, RNA was isolated from exponentially growing cultures of *R. palustris* TIE-1 strains (OD_660_ 0.2 for aerobic cultures grown on YP alone or supplemented with 1 mM succinate, acetate, benzoate, 4-hydroxybenzoate; for photoheterotrophic growth in FW medium supplemented with 1 mM succinate, acetate, benzoate, 4-hydroxybenzoate cells were harvested at OD_660_ of 0.2; for photoautotrophic growth on H_2_ cells were harvested at OD_660_ of 0.2; for photoautotrophic growth on Fe(II) cells were harvested when half of the added Fe(II) was oxidized). The anoxic cultures were harvested in a Coy anaerobic chamber and the aerobic samples were harvested on the bench top. Details of the qRT-PCR protocol can be found in the supporting information (the primers used are indicated in Table S5).

### Overexpression and purification of FixK from *E. coli*

*Escherichia coli* Rosetta (DE3) pLysS (Novagen, Gibbstown, NJ, USA) cells carrying the appropriate overexpression plasmid were grown in LB broth with 25 μg ml^−1^ chloramphenicol and 50 μg ml^−1^ ampicillin to mid-log phase at 37°C. The cells were then cold-shocked on ice for 15 min followed by induction with 1 mM IPTG and the cells were grown at 30°C for 24 h. The cultures were then pelleted at 4000 *g* and the cell pellets frozen at −80°C untill use. Details of the purification protocol can be found in the supporting information.

### Electrophoretic mobility shift assay

The DIG Gel Shift Kit, second generation (Roche, Indianapolis, IN, USA) was used as a non-radioactive way to perform electrophoretic mobility shift assays using the manufacturer's specifications. PCR was used to generate the DNA substrates (the primers used are indicated in Table S6) and the resulting products were gel purified using Wizard SV Gel and PCR Clean-Up System (Promega, Madison, WI, USA). Two substrates were designed to encompass the first (120 bp) and second (150 bp) consensus FixK binding sites as depicted in Fig. 6. The third substrate was designed to include both the first and second consensus FixK binding sites (247 bp) (Fig. 6). The control probe encompassing the Oct2A binding site (39 bp) was used to ascertain the specificity of FixK binding, which was provided in the DIG Gel Shift Kit, second generation (Roche, Indianapolis, IN, USA). The unlabelled probes were diluted as specified by the manufacturer and labelled with digoxygenin-11-dUTP (DIG) as specified. Details of the gel mobility-shift assays are provided in the supporting information.

### Other procedures

Fe(II) concentration was measured using the Ferrozine assay (Stookey, 1970). Total protein during growth on Fe(II)-NTA was measured using trichloroacetic acid (TCA) precipitation as follows: total protein in 2 ml of culture was precipitated using 500 μl 100% TCA. This mixture was incubated for 10 min at 4°C and spun at 14 000 r.p.m. for 30 min in a microcentrifuge at 4°C. The pellet was washed with 200 μl cold acetone at 14 000 r.p.m. for 10 min in a microcentrifuge at 4°C. The pellet was dried at 95°C for 10 min to remove residual acetone and resuspended in 50 μl 2.67 M guanidine HCl buffered with 100 mM Tris-Cl pH 8.0. The BCA (bichinchoninic acid) Protein Assay Kit was employed using the microtitre plate method for protein estimation as specified by the manufacturer following TCA precipitation with bovine serum albumin as control (Thermo Scientific, Waltham, MA, USA). Absorbance at 562 nm was measured using the Biotek Synergy 4 microtitre plate reader.

## References

[b1] Batut J, Daveranmingot ML, Jacobs MDJ, Garnerone AM, Kahn D (1989). FixK, a gene homologous with Fnr and Crp from *Escherichia coli,* regulates nitrogen-fixation genes both positively and negatively in *Rhizobium meliloti*.

[b2] Beatty CM, Browning DF, Busby SJ, Wolfe AJ (2003). Cyclic AMP receptor protein-dependent activation of the Escherichia coli acsP2 promoter by a synergistic class III mechanism.

[b3] Bobik C, Meilhoc E, Batut J (2006). FixJ: a major regulator of the oxygen limitation response and late symbiotic functions of Sinorhizobium meliloti.

[b4] Bose A, Metcalf WW (2008). Distinct regulators control the expression of methanol methyltransferase isozymes in Methanosarcina acetivorans C2A.

[b5] Browning DF, Busby SJ (2004). The regulation of bacterial transcription initiation.

[b6] Conlan S, Lawrence C, McCue LA (2005). Rhodopseudomonas palustris regulons detected by cross-species analysis of alpha-proteobacterial genomes.

[b7] Corcoran L, Shore P (2000). Transcription factors in B-cell development and function.

[b8] Cosseau C, Batut J (2004). Genomics of the ccoNOQP-encoded cbb3 oxidase complex in bacteria.

[b9] Croal LR, Gralnick JA, Malasarn D, Newman DK (2004). The genetics of geochemistry.

[b10] Crosson S, McGrath PT, Stephens C, McAdams HH, Shapiro L (2005). Conserved modular design of an oxygen sensory/signaling network with species-specific output.

[b11] Ehrenreich A, Widdel F (1994). Anaerobic oxidation of ferrous iron by purple bacteria, a new type of phototrophic metabolism.

[b12] Elbehti A, Brasseur G, Lemesle-Meunier D (2000). First evidence for existence of an uphill electron transfer through the bc(1) and NADH-Q oxidoreductase complexes of the acidophilic obligate chemolithotrophic ferrous ion-oxidizing bacterium Thiobacillus ferrooxidans.

[b13] Escolar L, Perez-Martin J, de Lorenzo V (1999). Opening the iron box: transcriptional metalloregulation by the Fur protein.

[b14] Feniouk BA, Junge W (2009). Proton translocation and ATP synthesis by the F_0_F_1_-ATPase of purple bacteria.

[b15] Gilles-Gonzalez MA, Gonzalez G (2005). Heme-based sensors: defining characteristics, recent developments, and regulatory hypotheses.

[b16] Green J, Baldwin ML (1997). HlyX, the FNR homologue of Actinobacillus pleuropneumoniae, is a [4Fe-4S]-containing oxygen-responsive transcription regulator that anaerobically activates FNR-dependent class I promoters via an enhanced AR1 contact.

[b18] Green J, Irvine AS, Meng W, Guest JR (1996). FNR-DNA interactions at natural and semi-synthetic promoters.

[b17] Green J, Baldwin ML, Richardson J (1998). Downregulation of Escherichia coli yfiD expression by FNR occupying a site at -93.5 involves the AR1-containing face of FNR.

[b19] Hantke K (2001). Iron and metal regulation in bacteria.

[b20] Hartman H, Cohen Y, Castenhole RW, Halvorson HO (1984). The evolution of photosynthesis and microbial mats: a speculation on the banded iron formations.

[b22] Heising S, Schink B (1998). Phototrophic oxidation of ferrous iron by a Rhodomicrobium vannielii strain.

[b21] Heising S, Richter L, Ludwig W, Schink B (1999). Chlorobium ferrooxidans sp nov., a phototrophic green sulfur bacterium that oxidizes ferrous iron in coculture with a ‘Geospirillum’ sp strain.

[b23] Jiao Y, Newman DK (2007). The pio operon is essential for phototrophic Fe(II) oxidation in Rhodopseudomonas palustris TIE-1.

[b24] Jiao YYQ, Kappler A, Croal LR, Newman DK (2005). Isolation and characterization of a genetically tractable photoautotrophic Fe(II)-oxidizing bacterium, Rhodopseudomonas palustris strain TIE-1.

[b25] Johnston AW, Todd JD, Curson AR, Lei S, Nikolaidou-Katsaridou N, Gelfand MS, Rodionov DA (2007). Living without Fur: the subtlety and complexity of iron-responsive gene regulation in the symbiotic bacterium Rhizobium and other alpha-proteobacteria.

[b26] Khan SR, Gaines J, Roop RM, Farrand SK (2008). Broad-host-range expression vectors with tightly regulated promoters and their use to examine the influence of TraR and TraM expression on Ti plasmid quorum sensing.

[b27] Larimer FW, Chain P, Hauser L, Lamerdin J, Malfatti S, Do L (2004). Complete genome sequence of the metabolically versatile photosynthetic bacterium Rhodopseudomonas palustris.

[b30] McClure WR (1985). Mechanism and control of transcription initiation in prokaryotes.

[b28] Main-Hester KL, Colpitts KM, Thomas GA, Fang FC, Libby SJ (2008). Coordinate regulation of Salmonella pathogenicity island 1 (SPI1) and SPI4 in Salmonella enterica serovar typhimurium.

[b29] Maruyama IN, Rakow TL, Maruyama HI (1995). cRACE – a simple method for identification of the 5'-end of messenger RNAs.

[b32] Mesa S, Ucurum Z, Hennecke H, Fischer HM (2005). Transcription activation in vitro by the Bradyrhizobium japonicum regulatory protein FixK2.

[b31] Mesa S, Hauser F, Friberg M, Malaguti E, Fischer HM, Hennecke H (2008). Comprehensive assessment of the regulons controlled by the FixLJ-FixK2-FixK1 cascade in Bradyrhizobium japonicum.

[b33] Miller JH (1992). Experiments in Molecular Genetics.

[b34] Nellen-Anthamatten D, Rossi P, Preisig O, Kullik I, Babst M, Fischer HM, Hennecke H (1998). Bradyrhizobium japonicum FixK2, a crucial distributor in the FixLJ-dependent regulatory cascade for control of genes inducible by low oxygen levels.

[b35] Oda Y, Larimer FW, Chain PS, Malfatti S, Shin MV, Vergez LM (2008). Multiple genome sequences reveal adaptations of a phototrophic bacterium to sediment microenvironments.

[b36] Poulain AJ, Newman DK (2009). Rhodobacter capsulatus catalyzes light-dependent Fe(II) oxidation under anaerobic conditions as a potential detoxification mechanism.

[b37] Rey FE, Harwood CS (2010). FixK, a global regulator of microaerobic growth, controls photosynthesis in Rhodopseudomonas palustris.

[b38] Schleyer M, Bakker EP (1993). Nucleotide sequence and 3'-end deletion studies indicate that the K(+)-uptake protein kup from Escherichia coli is composed of a hydrophobic core linked to a large and partially essential hydrophilic C terminus.

[b39] Stevens JM, Uchida T, Daltrop O, Ferguson SJ (2005). Covalent cofactor attachment to proteins: cytochrome c biogenesis.

[b40] Stookey LL (1970). Ferrozine – a new spectrophotometric reagent for iron.

[b41] Straub KL, Rainey FA, Widdel F (1999). Rhodovulum iodosum sp. nov, and Rhodovulum robiginosum sp. nov., two new marine phototrophic ferrous-iron-oxidizing purple bacteria.

[b42] Weber KA, Achenbach LA, Coates JD (2006). Microorganisms pumping iron: anaerobic microbial iron oxidation and reduction.

[b43] Widdel F, Schnell S, Heising S, Ehrenreich A, Assmus B, Schink B (1993). Ferrous iron oxidation by anoxygenic phototrophic bacteria.

[b44] Zhang R, Evans G, Rotella FJ, Westbrook EM, Beno D, Huberman E (1999). Characteristics and crystal structure of bacterial inosine-5'-monophosphate dehydrogenase.

