# Supplementary material for: Regulation of the phototrophic iron oxidation (pio) genes in Rhodopseudomonas palustris TIE-1 is mediated by the global regulator, FixK
Source: Mol Microbiol. 2010 Oct 28;79(1):63–75. doi: 10.1111/j.1365-2958.2010.07430.x (PMC3050613; doi:10.1111/j.1365-2958.2010.07430.x)
Supplement: Supplementary file 1 [file mmi7430-0063-SD1.pdf]

Table S1. Growth characteristics of *Rhodopseudomonas palustris* TIE-1 WT and *ΔfixK* during aerobic and anaerobic growth[a].

Strain	Succinate		Acetate		Benzoate		4-Hydroxybenzoate		Hydrogen	
	Aerobic	Anaerobic	Aerobic	Anaerobic	Aerobic	Anaerobic	Aerobic	Anaerobic	Aerobic	Anaerobic
**Generation time (h)**										
WT	3.8 ± 0.5	3.7 ± 0.2	4.7 ± 1.2	1.8 ± 0.1	4.8 ± 0.1	4.0 ± 0.6	4.9 ± 0.6	2.8 ± 0.1	ND	2.7 ± 0.3
ΔfixK	6.1 ± 0.9	9.4 ± 0.1	3.0 ± 0.1	8.0 ± 0.3	8.2 ± 1.3	7.4 ± 0.5	5.7 ± 1.1	6.9 ± 0.5	ND	7.9 ± 0.4
**Lag time (h)**										
WT	15.6 ± 2.2	20.9 ± 1.4	16.6 ± 1.7	8.3 ± 0.1	19.6 ± 0.2	20.1 ± 3.1	12.1 ± 3.2	18.4 ± 0.8	ND	17.6 ± 1.8
ΔfixK	16.3 ± 1.3	52.9 ± 0.8	19.3 ± 0.2	36.9 ± 1.5	19.6 ± 0.8	49.0 ± 3.6	13.6 ± 2.0	45.6 ± 3.5	ND	52.6 ± 2.3

a. Growth was measured as indicated in *Experimental procedures*; lag time represents the time required to reach 0.1 OD_{660}. Values represent the average and standard deviation of triplicate measurements. ND – Not determined, as aerobic growth could not be performed with hydrogen.

Table S2. Strains and plasmids used in the study.

Strain	Genotype and Use	Reference
E. coli DH10B	F^- *endA1 recA1 galE15 galK16 nupG rpsL ΔlacX74 Φ80lacZΔM15 araD139 Δ(ara,leu)7697 mcrA Δ(mrr-hsdRMS-mcrBC)* λ^-. Used as standard cloning strain.	(Casadaban & Cohen, 1980)
E. coli S17-1/λpir	λpir lysogen of strain S17-1 [*thi pro hdsR hdsM+ recA*; chromosomal insertion of RP4-2 (Tc::Mu Km::Tn7)]. Used as a conjugative donor strain.	(de Lorenzo *et al.*, 1993)
R. palustris TIE-1	Isolated as a phototrophic iron oxidizer from Woods Hole, MA. Served as WT strain.	(Jiao *et al.*, 2005)
AB8	*R. palustris* TIE-1 with a P*pio-lacZ* fusion inserted at the *glmUSX-recG* locus.	This study
AB10	*ΔfixK* derivative of AB8	This study
AB15	AB10 complemented with WT *fixK* cloned into pSRKGm	This study
AB20	AB10 complemented with WT NtermHis$_6$-*fixK* cloned into pSRKGm	This study
**Plasmid**	**Description and/or Construction**	**Reference**
pBBR1MCS-5	Broad host range mobilizable plasmid; Gm^r, lacZalpha, 4.8 kb	(Kovach *et al.*, 1995)
pJQ200KS	Mobilizable suicide vector; *sacB*, Gm^r	(Quandt & Hynes, 1993)
pSRKGm	Complementation plasmid modified from pBBR1MCS-5; Gm^r	(Khan *et al.*, 2008)
pCR®2.1-TOPO®	Commercial 3.9 kb TOPO-TA cloning vector; Km^r, Ap^r, lacZalpha	Invitrogen, Carlsbad, CA
pET-11a	Commercial *E. coli* overexpression plasmid; Ap^r	Novagen, Gibbstown, NJ
pUC18-mini-Tn7-Gm-*lacZ*	Source of *lacZ* gene; Gm^r	(Choi & Schweizer, 2006)

pAB300	Fusion PCR was used to remove an internal *Nde*I site in the *lacZ* gene from pUC18-mini-Tn7-Gm-*lacZ* using primers lacZfor and lacZNdeI-rev for PCR1; lacZrev and lacZNdeI-for were used for PCR2. *R. palustris* TIE-1 genomic DNA was used as template. PCR3 was performed using lacZfor and lacZrev with PCR1 and PCR2 as templates. This resulted in a lacZ gene without an internal *Nde*I site that was then cloned into *Nco*I-*EcoR*I digested pBBR1MCS-5. This *lacZ* gene however had a Phe to Leu mutation at position 1008.	This study
pAB301	Fusion PCR was used to remove the revert the Phe to Leu mutation at position 1008 of the *lacZ* gene in pAB300 using primers lacZmp(F)for and lacZrev for PCR1; lacZmp(F)rev and lacZfor were used for PCR2. pAB300 was used as template. PCR3 was performed using lacZfor and lacZrev with PCR1 and PCR2 as templates. This PCR was then cloned into the *Nco*I-*EcoR*I digested pAB300.	This study
pAB307	The promoter region of *pioABC* operon of *Rhodopseudomonas palustris* TIE-1 was amplified using primers PpioTIE1for and PpioTIE1rev and cloned into *Asc*I-*Nde*I digested pAB301.	This study
pAB314	Fusion PCR was used to amplify the 2 kb region surrounding the *glmUSX-recG* locus of *R. palustris* TIE-1 cloned into pJQ200KS. PCR1 was performed using TIE-1upfor and TIE-1fusionrev(new); PCR2 was performed using TIE-1dnrev and TIE-1fusionfor(new). *R. palustris* TIE-1 genomic DNA was used as template. PCR3 was performed using TIE-1upfor and TIE-1dnrev with PCR1 and PCR2 as templates. This PCR product has an internal *Nco*I site was then cloned into *Sph*I-*Sma*I digested pJQ200KS.	This study
pAB322	The Ppio^{TIE-1}-*lacZ* translational fusion was amplified from pAB307 using primers lacZforpartial(NcoI) and lacZrev(NcoI) and cloned into *Nco*I digested pAB314.	This study
pAB343	The putative first FixK binding site (BS1) was deleted using fusion PCR. PCR1 was performed using PpioTIE1for and PpioTIE1-FixK2BSrev. PCR2 was performed using PpioTIErev and PpioTIE1-FixK2BSfor. *R. palustris* TIE-1 genomic DNA was used as template. PCR3 was performed using PpioTIE1for and PpioTIE1rev with PCR1 and PCR2 as templates. This product was cloned into *Asc*I-*Nde*I digested pAB322.	This study

pAB404	The putative second FixK binding site (BS2) was deleted using fusion PCR. PCR1 was performed using PpioTIE1for and FixK2-BS2rev. PCR2 was performed using PpioTIErev and FixK2-BS2for. *R. palustris* TIE-1 genomic DNA was used as template. PCR3 was performed using PpioTIE1for and PpioTIE1rev with PCR1 and PCR2 as templates. This product was cloned into *Asc*I-*Nde*I digested pAB322.	This study
pAB337	Fusion PCR was used to fuse the 1 kb upstream and 1 kb downstream region of Rpal_4729 gene of *R. palustris* TIE-1 (FixK). PCR1 was performed using primers DRpal_4729for and DRpal_4729fusrev(new). PCR2 was performed using primers DRpal_4729rev2 and DRpal_4729fusfor(new). *R. palustris* TIE-1 genomic DNA was used as template. PCR3 was perfomed using primers DRpal_4729for and DRpal_4729rev2 with PCR1 and PCR2 as templates. This product was then cloned into *Spe*I-*Not*I digested pJQ200KS.	This study
pAB363	For complementation of *ΔfixK* (AB10) with native *fixK*, Rpal_4729 was amplified using FixK2pSRKGmfor(NdeI) and FixK2(SpeI)rev with *R. palustris* TIE-1 genomic DNA as template. This PCR product was cloned into *Nde*I-*Spe*I digested pSRKGm.	This study
pAB368	For affinity purification using Ni-NTA affinity chromatography an N-terminal 6X-Histidine tagged version of *fixK* from *R. palustris* TIE-1 was amplified using primers and FixK2TIE1NtermHisfor(NdeI)and FixK2rev(BamHI) with *R. palustris* TIE-1 genomic DNA as template. This PCR product was then cloned into *Nde*I-*Bam*HI digested pET-11a.	This study
pAB408	N-terminal 6X-Histidine tagged version of *fixK* from *R. palustris* TIE-1 was reamplified from pAB368 using primers FixK2TIE1NtermHisfor(NdeI) and FixK2(SpeI)rev. This PCR product was then cloned into the NdeI-SpeI digested pSRKGm.	This study

Table S3. Primers used in the study for plasmid construction.

Primer	Sequence
lacZfor	CTCGAGCCATGGTAAACCGATACAATTAAAGGCTCCTTTTGGAGCCTTTTTTTTTGGAGGGCGCGCCAAGCTTCATATGACCAT GATTACGGATTCACTG
lacZNdeI-rev	CGCCACCAATCCCCATGTGGAAACCGTCGATATTC
lacZrev(SpeI)	GGCGCGCCACTAGTAAAAAACCCCTCAAGACCCGTTTAGAGGCCCCAAGGGGTTATTATTTTTGACACCAGACCAACTG
lacZNdeI-for	GAATATCGACGGTTTCCACATGGGGATTGGTGGCG
lacZmp(F)for	GTCAGTATCGGCGGAGTTCCAGCTGAGCG
lacZmp(F)rev	CGCTCAGCTGGAACTCCGCCGATACTGAC
PpioTIE1for	GGCGCGCCAAGCTTGATTGCGAGTCGTCCTGTTGCACAACG
PpioTIE1rev	GGCGCGCCCATATGGTCCCCCGCAAGTGCTCAAAGC
TIE-1upfor	GGCGCGCCGCATGCCACACCGGCAGGTTGTTGATGGCTG
TIE-1fusionrev(new)	CGGGTTAGTTACCACGCGTCATTACTAGTTCGCGACCATGGCTACCCGACCTTGTCCGGCGCCTTTTC
TIE-1dnrev	ACTAGTCCCGGGCGAGATCGATTTTCTGGTCGGCAC
TIE-1fusionfor(new)	GAAAAGGCGCCGGACAAGGTCGGGTAGCCATGGTCGCGAACTAGTAATGACGCGTGGTAACTAACCCG
lacZforpartial(NcoI)	CTCGAGCCATGGTAAACCGATACAATTAAAGGCTC
lacZrev(NcoI)	GGCGCGCCCCATGGAAAAAACCCCTCAAGACCCGTTTAGAG
PpioTIE-1-FixK2BSrev	GTGGTCGACTAACTCTTGATTGCCCTGTGCGCAATGCAGC

PpioTIE-1-FixK2BSfor	GCTGCATTGCGCACAGGGCAATCAAGAGTTAGTCGACCAC
FixK2-BS2rev	TCTGCTGCGTATGCTAACAGATGTGAATAAATACGCGTTTTAGTACGTTGTGTGAC
FixK2-BS2for	GTCACACAACGTACTAAAACGCGTATTTATTCACATCTGTTAGCATACGCAGCAGA
DRpal_4729for	GGCGCGCCACTAGTTCGGACTTCATCGAGAAGCCATTCG
DRpal_4729fusrev(new)	CGATCCGATGAAAGAGATGCTCTCTAGATTACTGGCCATCTCCTTTGATC
DRpal_4729rev2	ACTAGTGCGGCCGCGATACTTGGTCGCCGAATACACCACGATC
DRpal_4729fusfor(new)	GATCAAAGGAGATGGCCAGTAATCTAGAGAGCATCTCTTTCATCGGATCG
FixK2pSRKGmfor(NdeI)	GGCGCGCCCATATGCTGAACCAGTCGCTCAGGACG
FixK2(SpeI)rev	GGCGCGCCACTAGTTTAGGCGTCGAGATTGCGAAGC
FixK2TIE1NtermHisfor(NdeI)	GGCGCGCCCATATGGGCAGCAGCCATCATCATCATCATCACAGCAGCGGCCTGGTGCCGCGCGGCAGCCACATGCTGAACCAGT CGCTCAGGAC
FixK2rev(BamHI)	ACTAGTGGATCCTTAGGCGTCGAGATTGCGAAGCTG
delFixK2upchkfor	GACACCGGCAGCGGTCTGC
delFixK2upchkrev	CAGAAGAGGCCACCATTGCTG
delFixK2dnchkfor	GATATGGAGCGAAATACCTCG
delFixK2dnchkrev	GCGAGGATGTCGTCGATG

Table S4. Primers used in the study for RACE.

Primer	Purpose	Sequence
pioARACErev1	5´RLM-RACE	ACCAGCGCATTGACCGAC
pioARACErev2	5´RLM-RACE, cRACE	CAGCGACGAGATCCAGCTC
PpioTIE1rev	5´RLM-RACE	GGCGCGCCCATATGGTCCCCCGCAAGTGCTCAAAGC
5′RACEouter	5′RLM- RACE	GCTGATGGCGATGAATGAACACTG
WNp213	cRACE	GTCTCGTTAGCTCGCTGGATCCTA 3´Inverted T
WNp210	cRACE	TAGGATCCAGCGAGCTAACGAGAC
M13 Forward (-20)	Sequencing	GTAAAACGACGGCCAG
M13 Reverse	Sequencing	CAGGAAACAGCTATGAC

Table S5. Primers used in the study for qRT-PCR.

Primer name	Sequence
Rpal_4713for	CTTCCTGGTCGGTTGGC
Rpal_4713rev	AGGCTGACGGTTTTAGTGAC
Rpal_3436for	ACATCTTCGGCGTGCTG
Rpal_3436rev	GTACTTCACCGTCACCAGC
Rpal_4015for	GGCAGGACCAAATCGCAG
Rpal_4015rev	GTTGATCGAGCAGTTGATTGG
Rpal_2453or	GATGACATCACGCCGGAG
Rpal_2453rev	AACCCGAGCACGTTGAC
Rpal_1413for	CATCGTCGGCATCGTCAG
Rpal_1413rev	ATCAGCTCCAGTGTGATTGAG
Rpal_0922for	ACATCACAGGCAAGAACGAAG
Rpal_0922rev	TGGTGAAGATGAAGCCCG
Rpal_0020for	GCATCGTCCTCTACATCTCG
Rpal_0020rev	ACGCCACATCAGACCTTG
Rpal_4994for	TGACCAAGTTTCTGACCGC
Rpal_4994rev	ACACGAGGTTGAATGCCG
Rpal_4717for	CTGACCTCGTTGACTTACCAG
Rpal_4717rev	CAGCACCATGATCGGGATC
Rpal_1280for	GCAGCTTCGTGTTCAACG
Rpal_1280rev	CGCGACGTTTTGGATCAC
Rpal_1868for	TTCGATCCGAGCTTCAACAG
Rpal_1868rev	GGATCACTCTGTCGGTTTCG
Rpal_1869for	TCCACCGGCTGACCTATC
Rpal_1869rev	GTGCCCTCTTCCTTGTAGC
Rpal_2582for	CCGCAAACGATCACACTTATG
Rpal_2582rev	GATGCCGCAACCGAATC
Rpal_2583for	ACACCCTCAATCACTTCGC
Rpal_2583rev	AATAGGTCTTGCTGCCATCG
Rpal_1206for	TGCATTGGATTCCGATCGTC
Rpal_1206rev	GCGACCAGGAACGAGAC
Rpal_1207for	CGACGCTCACCTTTCTTGAAG
Rpal_1207rev	ACATAGAGCTTCTTGGCGC
Rpal_1691for	GCTGGGTTTGTTCCGATTG
Rpal_1691rev	GGATGTGTTTCTTGCGGATTC
Rpal_1692for	ATCGATAATCACCAGTCGCG
Rpal_1692rev	CCGTTGAGCACCCACAG
Rpal_1412for	GGTCCACTGCTGATACTCATG
Rpal_1412rev	GTCGAAGGTGAGATACGCTG
Rpal_4729for	GCCTCGGAATTCACCTACAAG
Rpal_4729rev	GATAGACGTATTCGGACGGC
Rpal_2130for	GAATTCGAGCGTGCGGT
Rpal_2130rev	GAGGCCGAGGAAGTAGC
pioAqRTPCRfor	AAATTTCGACGACACCATCGA
pioAqRTPCRrev	CTTGGCGGCGAGGATCT
pioBqRTPCRfor	TCGCGCCAGGGTTCTATG
pioBqRTPCRrev	TTCCAGTAGGTGCCGTCCTT
pioCqRTPCRfor	ACGCCCAGGTCACCAAGA
pioCqRTPCRrev	GTTGGGCGACTCCTGATAGC

Table S6. Primers used in the study for PCR to generate DNA substrates for EMSA.

Primer name	Sequence
FixK2BS1for	CCGCAGAGCGCCTCCATCCAG
FixK2BS1rev	GCAGAACTAACAGGTCTGCGGTG
FixK2BS2for	CACCGCAGACCTGTTAGTTCTGC
FixK2BS2rev	CCTGCATCGCGACGCTTGC

Detailed description of putative FixK upregulated during anaerobic growth.

The putative FixK targets that were upregulated during anaerobic growth included genes involved in gene regulation, photosynthesis, respiration and transport-related functions. The regulatory proteins are AadR (Rpal_4713), a CRP/FNR family regulator that has been shown to be important for anaerobic degradation of benzoate and 4-hydroxybenzoate in *R. palustris* CGA009 (Dispensa *et al.*, 1992); predicted CRP/FNR family gene (Rpal_1280), an *Irr* homolog (Rpal_2583) and a PadR family protein (Rpal_1207). Proteins belonging to the PadR family are repressors of the *padA* gene that encodes a detoxifying decarboxylase of phenolic acids such as p-coumarate (Barthelmebs *et al.*, 2000, Gury *et al.*, 2004). Interestingly, such compounds can be utilized by *R. palustris* CGA009 and likely by *R. palustris* TIE-1 (Pan *et al.*, 2008). The following genes important for photosynthesis were upregulated during photosynthetic growth: *hemO* (Rpal_0922), *bchD* (Rpal_1692) and *cycH*. HemO is predicted to be 5-aminolevulinate synthase, an important early enzyme in the porphyrin and bacteriochlorophyll biosynthetic pathway (Evans *et al.*, 2008). BchD is a protein important in porphyrin biosynthesis that precedes bacteriochlorophyll biosynthesis (Willows & Kriegel, 2008). CycH is important in cytochrome C biogenesis, thus playing a key role in both photosynthesis and respiration (Lang *et al.*, 1996). Genes involved in respiration that were induced include: *ccoN(OQP)*, encoding the *cbb3* cytochrome C terminal oxidase with high affinity for O_2 (Pitcher *et al.*, 2002), a small hypothetical gene near *ccoG*, and a cytochrome C accessory protein (Preisig *et al.*, 1996). The upregulated genes

encoding transport related proteins were: *ompW*, an outer membrane porin homolog (Rpal_4994)(Lou *et al.*, 2009); *osmY*, a putative periplasmic osmotic shock gene (Rpal_1868)(Yim & Villarejo, 1992); and a predicted ORF in an operon with a heavy metal transporter (Rpal_2582)(Sitthisak *et al.*, 2007). In addition, a small predicted ORF encoding a protein likely involved in siderophore biosynthesis (Rpal_4015) and Rpal_1412, a signal peptide containing ORF with transmembrane domains were also highly upregulated under anoxic photosynthetic conditions. Overall these data show that *R. palustris* TIE-1 responds dramatically to a shift from aerobic chemoheterotrophic to anoxic phototrophic growth changing expression of a number of potential FixK target genes.

Detailed experimental procedures.

Construction of a single integration system for *R. palustris* TIE-1. The intergenic region of the operon Rpal_2933-2935 (*glmUS* homolog followed by a ORF of unknown function called gene *glmX* here) and Rpal_2936 (*recG* homolog) was chosen as a permissive locus based on the usage of this locus as the insertion site for the Mini-Tn7 transposon in diverse bacteria (Koch *et al.*, 2001). As *R. palustris* TIE-1 does not harbor this commonly found Tn7 insertion site, we devised a homologous recombination based single integration system that was based on the pJQ200KS counter-selection plasmid used for construction of mutants in *R. palustris* TIE-1 (Jiao & Newman, 2007). A 2 kb region surrounding the *glmUSX-recG* intergenic region was cloned into pJQ200KS giving rise to pAB314. A unique *Nco*I site was incorporated in the middle of this 2kb region such that the DNA to be incorporated could be cloned into pAB314. This system was later adapted to integrate *lacZ* translational fusions into the chromosome of *R. palustris* TIE-1 as described below. The pAB314-derived plasmids can be integrated on the *R. palustris* TIE-1 chromosome using selection on gentamicin to form a merodiploid. Counterselection using sucrose results in segregation of the merodiploid to give rise to either strains that have the *lacZ* fusion on the chromosome or WT *R. palustris* TIE-1. PCR screening was performed to confirm the correct strain was constructed.

Construction of a *lacZ* reporter system for *R. palustris* TIE-1. The *lacZ* gene that encodes β-galactosidase of *E. coli* was amplified from pUC18-mini-Tn7-Gm-*lacZ*, engineering the fd terminator in front and the T7 terminator at the end of the gene. This PCR product was cloned into the *Nco*I and *Eco*RI sites of pBBR1-MCS5 such that an *Nde*I site was engineered to overlap with the ATG of *lacZ*. In front of this *Nde*I site, the restriction site for a rare 8 bp cutter *Asc*I was engineered resulting in pAB301 (Figure S6). The inherent Plac promoter in pBBR1-MCS5 was deleted during this construction resulting in a promoter-less *lacZ* plasmid. *Asc*I-*Nde*I sites can be used for cloning a desired promoter region and will result in the formation of a translational fusion. As the pBBR1 plasmid and its derivatives are broad host range plasmids, pAB301 can be used as a plasmid of general use. The *lacZ* system was designed as a cassette system so that it can be easily sub-cloned into other pBBR1 derivatives in case other selectable markers are desired.

Determination of transcription start sites. 5'RLM-RACE. Total RNA was isolated as described in the *Experimental procedures*. Reverse transcription (RT) was performed using SuperScript III RNase H- reverse transcriptase (Invitrogen, Carlsbad, CA), according to the manufacturer's protocol using gene specific primers (Table S4). PCR amplification of the cDNA from the RT reaction was performed with Taq DNA polymerase (New England Biolabs, Ipswich, MA) as recommended. The PCR reactions were run on a 2% agarose gel stained with 5 μg/ml of ethidium bromide. Relevant bands were cut out and DNA from the bands was purified using the Wizard® SV Gel and PCR Clean-Up System

(Promega, Madison, WI). The bands that were specific to the TAP plus reaction represented the transcription start site and the bands common to the TAP plus and minus reactions represented processed sites. The PCR products were reamplified using a nested primer using Taq DNA polymerase as recommended (New England Biolabs, Ipswich, MA). The primers used for amplification are listed in Table S4. The products were then cloned into pCR®2.1-TOPO® using the TOPO-TA cloning kit as per the manufacturer's guidelines (Invitrogen, Carlsbad, CA). The PCR products were sequenced at the Biopolymers Laboratory in the Massachusetts Institute of Technology Center for Cancer Research.

cRACE. Total RNA was isolated as described in the *Experimental procedures* and contaminating chromosomal DNA was digested with TURBO DNA-free (Ambion, Austin, TX). Reverse transcription was performed using the Superscript II RNase H- reverse transcriptase (Invitrogen, Carlsbad, CA) as follows: 5 μg of the resulting RNA was combined with 20 pmols of the gene specific primer and 0.5 mM dNTPs. The resulting mixture was heated to 70°C for 10 min and immediately frozen on ice for at least 1 min. The first strand synthesis buffer, 10 mM DTT, 4 U/μl Rnasin (Promega, Madison, WI) were added to the above mixture as specified and heated to 42°C for 2 min. To this mixture Superscript II RNase H- reverse transcriptase was added and the reverse transcription was performed as specified. Following reverse transcription 0.1 U/μl Rnase H (Invitrogen, Carlsbad, CA) was added and the reaction mixture was incubated at 37°C for 20 min. The reaction mixture was then purified using the Wizard® SV

Gel and PCR Clean-Up System (Promega, Madison, WI) and eluted in 32 μl of nuclease free water. This cDNA was ligated to 100 pmols of WNp213 (a primer that has an inverted T on its 3′ end to prevent self-ligation) using T4 RNA ligase (Ambion, Austin, TX) in a 100 μl reaction. Ligations were performed in 25% polyethylene glycol 8000, 1 mM hexaammine cobalt (III) chloride, 0.1 mg/ml BSA and T4 RNA ligase buffer at 22°C for 16 hrs (a brown precipitate forms). This reaction was purified using the Wizard® SV Gel and PCR Clean-Up System (Promega, Madison, WI) and eluted in 20 μl nuclease free water. This eluate was used for PCR amplification using a nested gene specific primer, primer WNp210 and Taq DNA polymerase (New England Biolabs, Ipswich, MA). The resulting bands were separated on a 2% agarose gel and the desired bands were gel-eluted using the Wizard® SV Gel and PCR Clean-Up System (Promega, Madison, WI). The products were then cloned into pCR2.1-TOPO using the TOPO-TA cloning kit as per the manufacturer's guidelines (Invitrogen, Carlsbad, CA). The PCR products were sequenced at the Biopolymers Laboratory in the Massachusetts Institute of Technology Center for Cancer Research.

Measurement of β-galactosidase activity. For photoheterotrophic growth cultures were grown on YP medium and inoculated into FW medium at a 10^{-2} dilution supplemented with appropriate electron donors. For photoautotrophic growth on H_2, cultures were grown on YP medium and inoculated into FW medium at a 10^{-2} dilution with H_2/CO_2. For photoferrotrophic growth cultures were grown on FW medium at 10^{-2} dilution with H_2/CO_2 and inoculated into FW medium with Fe(II)/Fe(II)-NTA; at least two passages were performed on

Fe(II)/Fe(II)-NTA before β-galactosidase activity was determined. A 10 ml culture in the logarithmic phase of growth (OD_{660} 0.2 for aerobic cultures grown on YP alone or supplemented with 1 mM succinate, acetate, benzoate, 4-hydroxybenzoate; for photoheterotrophic growth in FW medium supplemented with 1 mM succinate, acetate, benzoate, 4-hydroxybenzoate cells were harvested at OD_{660} 0.2; for photoautotrophic growth on H_2 cells were harvested at OD_{660} 0.2; for photoautotrophic growth on Fe(II)-NTA cells were harvested OD_{660} 0.1; for photoautotrophic growth on Fe(II) cells were harvested when half of the added Fe(II) was oxidized) were harvested and resuspended in 200 μl of Z-buffer (0.06 M $Na_2HPO_4.2H_2O$, 0.04 M NaH_2PO_4, 0.01 M KCl, 0.001 M $MgSO_4$, 0.05 M β-mercaptoethanol, pH 7.0) and the cells were permeabilized by adding 20 μl chloroform and 10 μl 0.1% SDS and incubated at room-temperature for 5 min. The cells were then stored on ice and sonicated using a Fisher Scientific Model 550 Sonic Dismembrator for a total of 2 min at the amplitude of 3 (3 s pulses followed by 6 s rest). The extracts were then spun at 14,000 rpm in a microcentrifuge at 4°C for 10 min. 20 -100 μl of cell extract was mixed with the Z-buffer to achieve 900 μl volume. The reaction was started by adding 100 μl of a 4 mg/ml solution of *o*-nitrophenylgalactopyranoside dissolved in phosphate buffer (0.06 M $Na_2HPO_4.2H_2O$ and 0.04 M NaH_2PO_4). The rate of increase in the *A*420 due to *o*-nitrophenol formation was measured spectrophotometrically using a Beckman Coulter DU 800 Spectrophotometer. Activity was calculated with a molar extinction coefficient (ε) of 4500 $liters.mol^{-1}.cm^{-1}$ for *o*-nitrophenol at 420 nm. Protein estimation was routinely performed using the microtiter plate method

for the Biorad Protein Assay reagent (Biorad, Hercules, CA) using BSA as standard (Thermo Scientific, Waltham, MA). Absorbance at 595 nm was measured using the Biotek Synergy 4 microtiter plate reader. Reported values for β-galactosidase activity represent mean and standard error of 9 independent measurements.

Quantitative reverse-transcription PCR. 5-10 ml of appropriately grown cultures were added to 2 volumes of RNAprotect Bacteria Reagent (Qiagen, Valencia, CA), incubated for 5 min at room temperature, and centrifuged for 20 min at 5000 X *g*. RNA was isolated from the cell pellet using the RNeasy Mini Kit (Qiagen, Valencia, CA) with proteinase K and lysozyme treatment as specified by the manufacturer. RNA samples were treated with TURBO DNA-*free* DNase (Ambion, Austin, TX) to remove genomic DNA contamination. All RNA samples were stored at -80°C till further use. 400 ng of RNA was used in a 100 μl cDNA synthesis reaction using the iScript cDNA Synthesis Kit (Biorad, Hercules, CA). The cDNA (1 μl) was used as template for quantitative reverse-transcription-PCR (qRT-PCR) using the iTaq SYBR Green Supermix with Rox (Biorad, Hercules, CA) on the 7500 Fast Real-Time PCR System (Applied Biosystems, Foster City, CA). Primer Express v2.0 (Applied Biosystems, Foster City, CA) was used to design primers targeting the transcripts to be tested (Table S5). The final concentration of primers used was 200 nM in a reaction volume of 20 μl. The cycling parameters used were 95°C for 3 min, followed by 40 cycles of 95°C for 15 s and 60°C for 30 s. A final dissociation curve was performed for each reaction to ensure that a single product was amplified each time. The *clpX* and

recA genes of *R. palustris* TIE-1 were used as an internal controls for normalization (Dietrich *et al.*, 2006). The ΔΔCt method was used for calculating mRNA abundance. ΔCt values for each sample were calculated by subtracting the Ct value of the *clpX* gene from the Ct value of the target gene. The ΔΔCt value was calculated by subtracting the ΔCt value of a calibrator condition from the ΔCt value of the test sample. The fold difference was then calculated using the formula $2^{-\Delta\Delta Ct}$ according to the Guide to Performing Relative Quantitation of Gene Expression using Real-Time Quantitative PCR (Applied Biosystems, Foster City, CA). All samples were assayed as three biological replicates measured in triplicate and the cycle time determined automatically by the Real Time 7500 PCR software (Applied Biosystems, Foster City, CA).

Overexpression and purification of FixK from *E. coli*. The cell pellets of 500 ml cultures carrying overexpressed His_6-FixK protein was resuspended in 5 ml ice-cold 50 mM 4-(2-hydroxyethyl)-1-piperazineethanesulfonic acid (HEPES) (pH 7.0) buffer containing 0.5 M NaCl (1X Native binding buffer) with 1X Complete, EDTA-free, Protease Inhibitor Cocktail (Roche, Indianapolis, IN). Cells were broken 2X in a French pressure cell (20,000 psi) and centrifuged at 13,000 X *g* for 30 min to pellet cell debris and the resultant supernatant was passed over a Ni-NTA agarose (5 ml bed volume) (Qiagen, Valencia, CA) gravity flow column pre-equilibrated with 10 bed volumes of 1X Native binding buffer. Contaminating *E. coli* proteins were washed off the column in a stepwise fashion with buffers identical to 1X Native binding buffer, but with varying concentrations of imidazole (20, 50, and 100 mM; 10 bed volumes of each of the buffers was used for

washes). His_6-FixK protein was collected from the column after washing with 2.5 ml of buffer containing 500 mM imidazole. His_6-FixK after affinity chromatography was purified to apparent homogeneity, as ascertained by SDS-PAGE followed by Coomassie Brilliant Blue staining (Figure S5). High-purity His_6-FixK was desalted using disposable PD-10 Desalting columns with a 10 kDa molecular weight cut-off as per the manufacturer's guidelines (GE Healthcare, Piscataway, NJ) and the original buffer was exchanged with 50 mM HEPES (pH 7.0), 250 mM KCl, containing 50% glycerol, 1 mM dithiothreitol (DTT) and 0.1 mM ethylenediaminetetraacetic acid (EDTA).

Electrophoretic mobility shift assay (EMSA). DNA probes were designed as indicated in the *Experimental procedures*. The labeling efficiency of the probes was then determined as specified and 20 μl gel shift reactions were set up with 30 fmol of DIG labeled probe, desired amount of protein, 1 μg of poly [d(I-C)], 1 μg of poly L-lysine in binding buffer [20 mM HEPES, pH 7.6, 1 mM EDTA, 10 mM $(NH_4)_2SO_4$, 1 mM DTT, Tween 20 0.2% (w/v), 30 mM KCl]. The reactions were incubated at room temperature for 15 min and loaded immediately on a pre-run 8% 0.5 X Tris-Borate-EDTA (TBE) polyacrylamide gel run at 80 V at room temperature till the bromophenol blue dye reached ¾ of the gel. The gels were then electro-blotted onto positively charged nylon membranes (Roche, Indianapolis, IN) using a Biorad Mini Trans-Blot Electrophoretic Transfer Cell at 400 A for 30 min in 0.5 X TBE. Following electro-transfer the DNA probes were detected by immunoassay as specified by the manufacturer followed by chemiluminescence using Amersham Hyperfilm ECL films (GE Healthcare,

Piscataway, NJ). The exposed films were developed and fixed using a Kodak (CareStream) M35A X-Omat film processor.

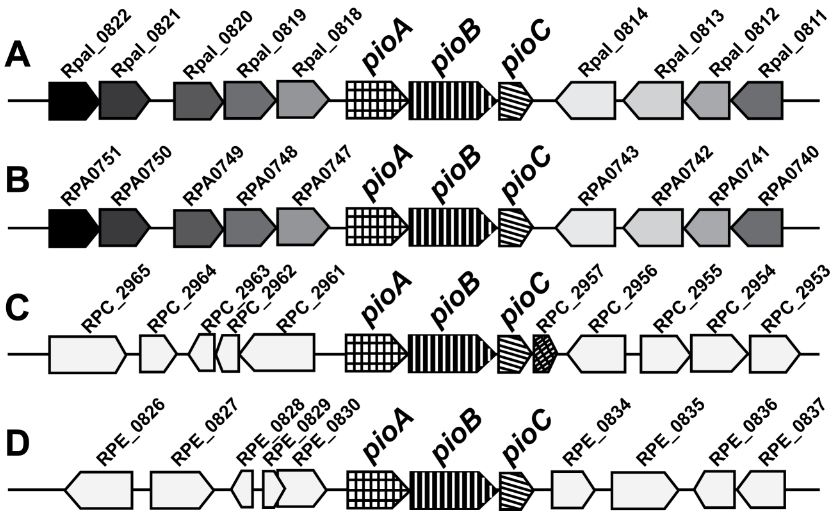

Figure S1. *pioABC* genetic locus organization in A: *R. palustris* TIE-1; **B**: *R. palustris* CGA009; **C**: *R. palustris* BisB18 and **D**: *R. palustris* BisA53. The genes depicted in grey are conserved in location in *R. palustris* TIE-1 and CGA009. The genes depicted in white are not conserved in location. The other genes are conserved in location and are color coded according to location.

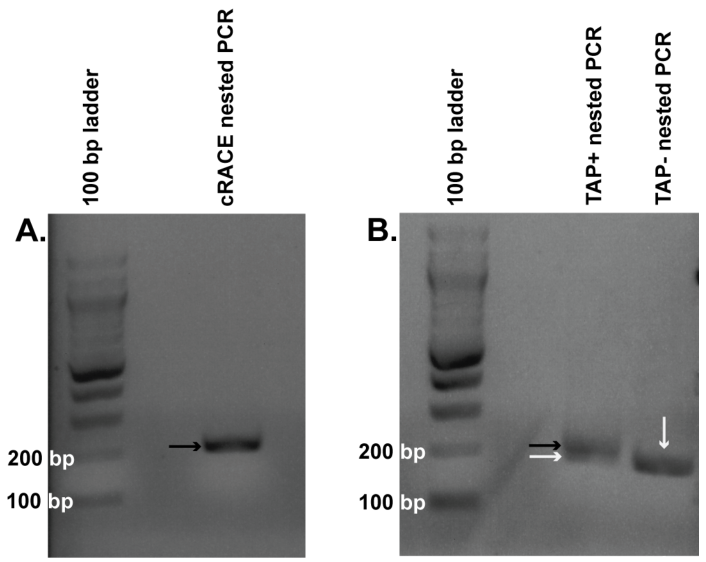

Figure S2. Nested PCR results for cRACE and 5'RLAM-RACE. Panel A. A representative 2% agarose gel with a 100 bp DNA marker and the 213 bp nested PCR product using primers PpioTIE-1rev and WNp210 (black arrow). **Panel B.** A representative 2% agarose gel with a 100 bp DNA marker and the 234 bp Tap+ (black arrow) and 209 bp TAP- (white arrow) nested PCR product using primers PpioTIE-1rev and 5'RACE outer primer. The 209 bp processed product can be seen as the lower band in the TAP+ lane. The relevant bands of the marker are shown. These experiments were performed using RNA isolated from photoferrotrophically grown cultures. Larger reactions of these PCRs were separated, gel eluted and cloned into pCR®2.1-TOPO® using the TOPO-TA cloning kit as per the manufacturer's guidelines (Invitrogen, Carlsbad, CA). The PCR products were sequenced at the Biopolymers Laboratory in the Massachusetts Institute of Technology Center for Cancer Research.

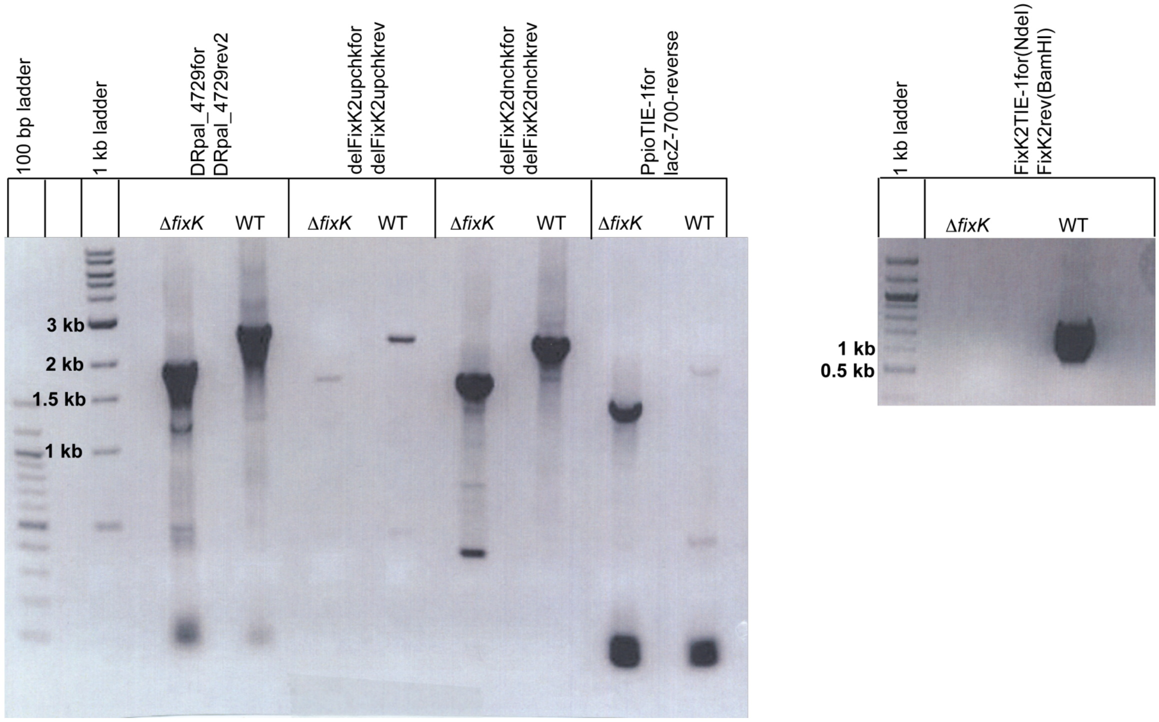

Figure S3. PCR to confirm the *ΔfixK* mutant of *R. palustris* TIE-1. Five independent PCR reactions were performed. The first PCR was used to confirm that the upstream and downstream regions of *fixK* cloned in to pJQ200KS was on the chromosome of the deletion strain. In the *ΔfixK* strain the product is be 1963 bp; the WT band is 2644 bp. The second PCR was to confirm the upstream locus of *ΔfixK*; mutant product is 1581 bp; WT product is 2271 bp. The third PCR was to confirm the downstream locus of *ΔfixK*: mutant product 1664 is bp; WT product is 2354 bp. The fourth PCR was to confirm that the strain had a *lacZ* gene; the mutant gives product 1102 bp long while WT does not yield any product. The fifth PCR was to confirm that *ΔfixK* did not yield a product for *fixK*; mutant does not give a product while WT gives a 702 bp product. Relevant bands in the DNA markers are indicated.

A. WT ΔfixK COM

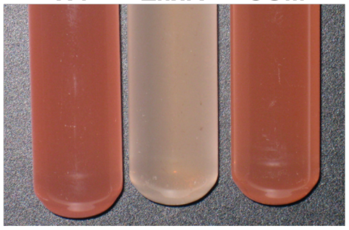

B. Fold-change in mRNA abundance during anaerobic growth on Fe vs. aerobic growth on succinate in WT, Δ*fixK* and complemented strains.

Strain	*pioA*	*pioB*	*pioC*	*fixK*
WT	1186.2±244.4	215.2±26.2	426.0±28.7	12.2±2.4
Δ*fixK*	11.4±2.4	5.8±1.0	2.2±0.2	ND
Δ*fixK fixK*-pSRKGm	79.8±9.9	34.4±1.5	4.6±0.6	7.2±0.6
Δ*fixK* NtermHis$_6$ *fixK*-pSRKGm	30.8±3.8	19.5±0.9	6.8±0.9	11.8±1.1

Figure S4. Complementation of Δ*fixK* using WT gene and NtermHis$_6$-tagged gene. A. WT *R. palustris* TIE-1 growing on FW succinate produces ample pigmentation under photoheterotrophic conditions while Δ*fixK* does not. Δ*fixK* complemented with native *fixK* had WT levels of pigmentation. **B.** qRT-PCR analysis comparing expression of *pioABC* and *fixK* in WT is compared to Δ*fixK* and strains complemented with the native *fixK* gene or the NtermHis$_6$-tagged version resulted in partial restoration of *pioABC* expression and full restoration of *fixK* expression.

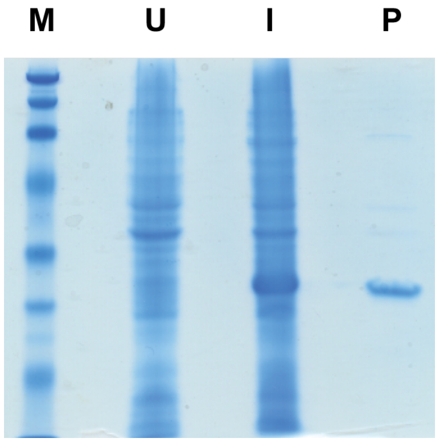

Figure S5. Purified His$_6$-FixK from *E. coli*. M – Broad range marker (Biorad, Hercules, CA). U – uniduced *E. coli* extract. I – induced *E. coli* extract. P – purified protein (27.6 kDa).

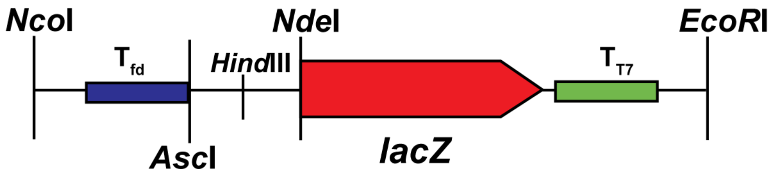

Figure S6. *lacZ* cassette made for use as translational fusion. The *lacZ* gene was flanked by fd terminator in front and T7 terminator at the end of the gene. An *Asc*I and *Nde*I site were engineered in the beginning of the *lacZ* gene to clone in any promoter region of interest in frame with the ATG start codon of *lacZ*.

References

Barthelmebs, L., B. Lecomte, C. Divies & J. F. Cavin, (2000) Inducible metabolism of phenolic acids in *Pediococcus pentosaceus* is encoded by an autoregulated operon which involves a new class of negative transcriptional regulator. *J Bacteriol* **182**: 6724-6731.

Casadaban, M. J. & S. N. Cohen, (1980) Analysis of gene control signals by DNA fusion and cloning in Escherichia coli. *J Mol Biol* **138**: 179-207.

Choi, K. H. & H. P. Schweizer, (2006) mini-Tn7 insertion in bacteria with single attTn7 sites: example Pseudomonas aeruginosa. *Nat Protoc* **1**: 153-161.

de Lorenzo, V., I. Cases, M. Herrero & K. N. Timmis, (1993) Early and late responses of TOL promoters to pathway inducers: identification of postexponential promoters in Pseudomonas putida with lacZ-tet bicistronic reporters. *J Bacteriol* **175**: 6902-6907.

Dietrich, L. E. P., A. Price-Whelan, A. Petersen, M. Whiteley & D. K. Newman, (2006) The phenazine pyocyanin is a terminal signalling factor in the quorum sensing network of *Pseudomonas aeruginosa*. *Mol Microbiol* **61**: 1308-1321.

Dispensa, M., C. T. Thomas, M. K. Kim, J. A. Perrotta, J. Gibson & C. S. Harwood, (1992) Anaerobic growth of *Rhodopseudomonas palustris* on 4-hydroxybenzoate is dependent on AadR, a member of the cyclic AMP receptor protein family of transcriptional regulators. *J Bacteriol* **174**: 5803-5813.

Evans, K., T. Georgiou, T. Hillon, A. Fordham-Skelton & M. Papiz, (2008) Bacteriophytochromes control photosynthesis in *Rhodopseudomonas palustris*. In: The Purple Phototrophic Bacteria. pp. 799-809.

Gury, J., L. Barthelmebs, N. P. Tran, C. Divies & J. F. Cavin, (2004) Cloning, deletion, and characterization of PadR, the transcriptional repressor of the phenolic acid decarboxylase-encoding *padA* gene of *Lactobacillus plantarum*. *Appl Environ Microbiol* **70**: 2146-2153.

Jiao, Y. & D. K. Newman, (2007) The *pio* operon is essential for phototrophic Fe(II) oxidation in *Rhodopseudomonas palustris* TIE-1. *J Bacteriol* **189**: 1765-1773.

Jiao, Y. Y. Q., A. Kappler, L. R. Croal & D. K. Newman, (2005) Isolation and characterization of a genetically tractable photoautotrophic Fe(II)-oxidizing bacterium, *Rhodopseudomonas palustris* strain TIE-1. *Appl Environ Microbiol* **71**: 4487-4496.

Khan, S. R., J. Gaines, R. M. Roop & S. K. Farrand, (2008) Broad-host-range expression vectors with tightly regulated promoters and their use to examine the influence of TraR and TraM expression on Ti plasmid quorum sensing. *Appl Environ Microbiol* **74**: 5053-5062.

Koch, B., L. E. Jensen & O. Nybroe, (2001) A panel of Tn7-based vectors for insertion of the *gfp* marker gene or for delivery of cloned DNA into Gram-negative bacteria at a neutral chromosomal site. *J Microbiol Methods* **45**: 187-195.

Kovach, M. E., P. H. Elzer, D. S. Hill, G. T. Robertson, M. A. Farris, R. M. Roop, 2nd & K. M. Peterson, (1995) Four new derivatives of the broad-host-range cloning vector pBBR1MCS, carrying different antibiotic-resistance cassettes. *Gene* **166**: 175-176.

Lang, S. E., F. E. Jenney, Jr. & F. Daldal, (1996) *Rhodobacter capsulatus* CycH: a bipartite gene product with pleiotropic effects on the biogenesis of structurally different c-type cytochromes. *J Bacteriol* **178**: 5279-5290.

Lou, H., K. Beis, J. H. Naismith & D. Larry, (2009) Bacterial membrane proteins: the new soluble proteins? In: Current Topics in Membranes. Academic Press, pp. 269-297.

Pan, C., Y. Oda, P. K. Lankford, B. Zhang, N. F. Samatova, D. A. Pelletier, C. S. Harwood & R. L. Hettich, (2008) Characterization of anaerobic catabolism of p-coumarate in *Rhodopseudomonas palustris* by integrating transcriptomics and quantitative proteomics. *Mol Cell Proteomics* **7**: 938-948.

Pitcher, R. S., T. Brittain & N. J. Watmough, (2002) Cytochrome cbb(3) oxidase and bacterial microaerobic metabolism. *Biochem Soc Trans* **30**: 653-658.

Preisig, O., R. Zufferey & H. Hennecke, (1996) The *Bradyrhizobium japonicum fixGHIS* genes are required for the formation of the high-affinity cbb3-type cytochrome oxidase. *Arch Microbiol* **165**: 297-305.

Quandt, J. & M. F. Hynes, (1993) Versatile suicide vectors which allow direct selection for gene replacement in gram-negative bacteria. *Gene* **127**: 15-21.

Sitthisak, S., L. Knutsson, J. W. Webb & R. K. Jayaswal, (2007) Molecular characterization of the copper transport system in *Staphylococcus aureus*. *Microbiology* **153**: 4274-4283.

Willows, R. D. & A. M. Kriegel, (2008) Biosynthesis of bacteriochlorophylls in purple bacteria. In: The Purple Phototrophic Bacteria. pp. 57-79.

Yim, H. H. & M. Villarejo, (1992) *osmY*, a new hyperosmotically inducible gene, encodes a periplasmic protein in *Escherichia coli*. *J Bacteriol* **174**: 3637-3644.
